# An Introductory Overview of Image-Based Computational Modeling in Personalized Cardiovascular Medicine

**DOI:** 10.3389/fbioe.2020.529365

**Published:** 2020-09-25

**Authors:** Thanh Danh Nguyen, Olufemi E. Kadri, Roman S. Voronov

**Affiliations:** ^1^Otto H. York Department of Chemical and Materials Engineering, Newark College of Engineering, New Jersey Institute of Technology, Newark, NJ, United States; ^2^UC-P&G Simulation Center, University of Cincinnati, Cincinnati, OH, United States; ^3^Department of Biomedical Engineering, Newark College of Engineering, New Jersey Institute of Technology, Newark, NJ, United States

**Keywords:** image-based modeling, personalized cardiovascular medicine, cardio electromechanics, hemodynamics, thrombogenesis, simulation, biomechanics, heart

## Abstract

Cardiovascular diseases account for the number one cause of deaths in the world. Part of the reason for such grim statistics is our limited understanding of the underlying mechanisms causing these devastating pathologies, which is made difficult by the invasiveness of the procedures associated with their diagnosis (e.g., inserting catheters into the coronal artery to measure blood flow to the heart). Likewise, it is also difficult to design and test assistive devices without implanting them *in vivo*. However, with the recent advancements made in biomedical scanning technologies and computer simulations, image-based modeling (IBM) has arisen as the next logical step in the evolution of non-invasive patient-specific cardiovascular medicine. Yet, due to its novelty, it is still relatively unknown outside of the niche field. Therefore, the goal of this manuscript is to review the current state-of-the-art and the limitations of the methods used in this area of research, as well as their applications to personalized cardiovascular investigations and treatments. Specifically, the modeling of three different physics – electrophysiology, biomechanics and hemodynamics – used in the cardiovascular IBM is discussed in the context of the physiology that each one of them describes and the mechanisms of the underlying cardiac diseases that they can provide insight into. Only the “bare-bones” of the modeling approaches are discussed in order to make this introductory material more accessible to an outside observer. Additionally, the imaging methods, the aspects of the unique cardiac anatomy derived from them, and their relation to the modeling algorithms are reviewed. Finally, conclusions are drawn about the future evolution of these methods and their potential toward revolutionizing the non-invasive diagnosis, virtual design of treatments/assistive devices, and increasing our understanding of these lethal cardiovascular diseases.

## Introduction: Image-Based Modeling of the Heart

Heart disease is the leading cause of death in the U.S., with one person dying from it every 37 s, or about 647,000 each year (i.e., 1 in every 4 deaths), and amounting to a $219 billion per year burden to the public health system (CDC, 2019). Understanding it is very difficult, because it is a complex interaction of biomechanics, electrophysiology and non-Newtonian hemodynamics. This is further complicated by the interaction with external medical devices (pacemakers, pumps, etc.) that are commonly implanted in order to assist a failing or dysfunctional heart. Moreover, the heart’s properties (e.g., shape, structure, stiffness, electrical conductivity) that play an important role in determining its pumping ability are patient specific. Finally, it is difficult to extract information about the physiological processes occurring in living hearts, due to its constant motion, and the fact that invasive probing can be life threatening. For these reasons, Image-Based Modeling (IBM) – a patient-specific experimentally constrained computational approach – is a lucrative way for gaining novel insight into the cardiovascular diseases and their treatments.

An illustrative example of the IBM’s usefulness is HeartFlow Inc. – a company located in California, United States with about 300 employees and backed by $467 million capital investment (Craft, 2019). Their application is the diagnosis of Coronary artery disease (CAD) – an impairment of blood flow in the arteries that supply the heart, due to cholesterol plaque buildup. The disease is one of the most misdiagnosed: a recent study, which included data from more than 1,100 U.S. hospitals, found that over half of the more than 385,000 patients with suspected CAD underwent an invasive coronary angiography (ICA) only to find out that they did not have the disease (Patel et al., 2014). This is bad because ICA is an invasive technique that in itself could lead to mortality, because it uses catheters inserted into the femoral (groin) or radial (wrist) arteries to measure pressure difference across a coronary artery stenosis in order to check the likelihood of a blockage’s presence.

**FIGURE 1:**
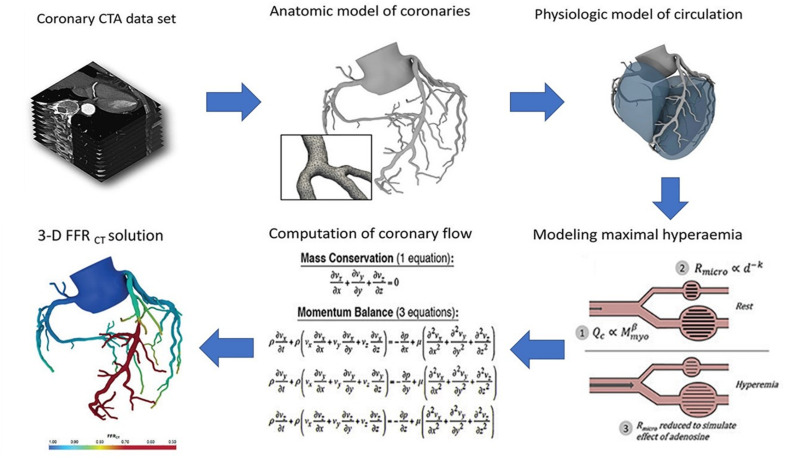
Process flow diagram outlining the image-based computational pipeline of the HeartFlow’s approach to calculating the fractional flow reserve computerized tomography (FFRCT). (Reproduced with permission from Nick Curzen, Professor of Interventional Cardiology/Consultant Cardiologist, University Hospital Southampton).

Conversely, HeartFlow calculates pressure differences virtually by simulating the blood flow through the patients’ own arteries, the structure of which is derived from a three-dimensional (3D) computerized tomography (CT) scan. This information is then used to calculate the fractional flow reserve (FFR), which is a statistic used to assess the hemodynamic significance of the stenosis by determining the ratio of the pressures before and after the narrowing. Therefore, this technology effectively serves as a non-invasive alternative to the ICA (Figure 1) (HeartFlow, 2019).

The HeartFlow’s method has been evaluated in four large prospective clinical trials, enrolling a total of more than 1,100 patients at major medical centers worldwide. It received the European Economic Area CE mark in 2011 and U.S. FDA clearance in August 2019 (i.e., it is currently commercially available in the U.S.) (FDA, 2019). To date, clinicians have used the HeartFlow approach for over 30,000 patients in the diagnosis of heart disease (Kim, 2019). Therefore, it serves as the most mature IBM application in the context of cardiovascular disease. Yet, it is also one of the simplest in that it does not include the heart itself, and the blood assumed a homogeneous (i.e., no cells) fluid. At the same time, more advanced models are coming online as well. Yet, they are relatively unknown outside of this niche field.

Although many excellent reviews already exist in the image-based heart-modeling area, most of them are focused on just one or two specific aspects: for example, image acquisition and processing (Weese et al., 2013; Lamata et al., 2014; Wang et al., 2015; Watson et al., 2018), hemodynamic flow simulations (Tang et al., 2010; Mittal et al., 2016; Quarteroni et al., 2017; Zhong et al., 2018); electrical conduction and stimulation modeling (Trayanova, 2011, 2012; Tobon-Gomez et al., 2013; Lopez-Perez et al., 2015; Rodriguez et al., 2015; Beheshti et al., 2016; Gray and Pathmanathan, 2018; Ni et al., 2018); tissue mechanics computations (Trayanova, 2011, 2012; Sun et al., 2014; Wang et al., 2015; Chabiniok et al., 2016; Niederer et al., 2019a,b), ventricular thrombosis (Mittal et al., 2016) and the use of models in diagnostic procedures (Tang et al., 2010; Trayanova, 2012). Whereas, the goal of this manuscript is to provide a brief introductory overview of the entire cardiovascular IBM for a non-expert audience, in order to increase the broader exposure of this exciting topic and its numerous potential applications: CAD, Arrhythmias, Heart Failure (HF), Left-Ventricular Assist Devices (LVAD) and Pathogenic Thrombosis/Embolism.

To that end, this review is organized as follows: Section “Methods: Literature Search” describes our literature search methods; Section “Background: Cardio Electromechanics and Hemodynamics” provides a brief background of the relevant cardiovascular physiology that explains how the tissue electromechanics and blood biology are interrelated *in vivo*; Section “Geometry Module” reviews how the model geometry is obtained using imaging, in order to establish a connection with the individual’s unique anatomy; Sections “Electrophysiology Module,” “Biomechanics Module,” “Simplified Hemodynamics Module,” and “Hemodynamics with Thrombogenesis Module” illustrate the mathematical formulation of the simulation modules used by these models, such as electrophysiology, biomechanics, simplified hemodynamics with and without thrombogenesis, respectively. Finally, Section “Summary and Conclusion” presents our summary and conclusions regarding the inputs and outputs of all cardiac modules as well as the directions that the field of personalized cardiovascular IBM is expected to go into.

## Methods: Literature Search

In order to provide a “big picture” snapshot overview of the image-based cardiovascular modeling and its potential penetration into the clinical sector, we gathered works from the recent (i.e., approximately the last 5 years) proceedings of the following meetings: Interagency Modeling and Analysis Group Consortium Meetings, National Institute for Mathematical and Biological Synthesis workshops, Personalized Medicine Coalition resources, International Workshop on Cancer Systems Biology meetings and conferences, Pacific Symposium on Biocomputing, Biomedical Engineering Society, American Institute of Chemical Engineers. Additionally, we performed manual searches with key words and terms including “image-based modeling of the heart,” “patient-specific cardiovascular modeling,” “cardio electrophysiology/biomechanics/electromechanics/hemodynamics image-based modeling” or “ventricular thrombosis modeling,” etc. for both research and review articles on databases, such as PubMed central, Web of Science, Research Gate, and Google Scholar. Additionally, we relied on the corresponding author’s own decade and a half long experience of working on biomedical image-based simulations. The obtained publication database was then screened by running citation reports to identify groups of researchers (typically led by a senior professor, who is joined by collaborators, postdocs, and students) that have established a track-record of being active within the various sub-areas of the cardiovascular modeling fields. Furthermore, to avoid bias (and to keep the work manageable) we tried to limit the literature sampling to just one most relevant publication from each of the groups. However, this was not always possible, because some of the researchers dominate their respective niches; and have published more than one article critical to our review.

## Background: Cardio Electromechanics and Hemodynamics

### Macroscopic Overview of How the Three Physics Are Coupled With Each Other

Before going into the details of the computational models, it is first important to understand the three types of coupled physics occurring in the heart: electrical signal conduction, biomechanics of the contraction and hemodynamics (which could also include clot formation and embolism).

**FIGURE 2:**
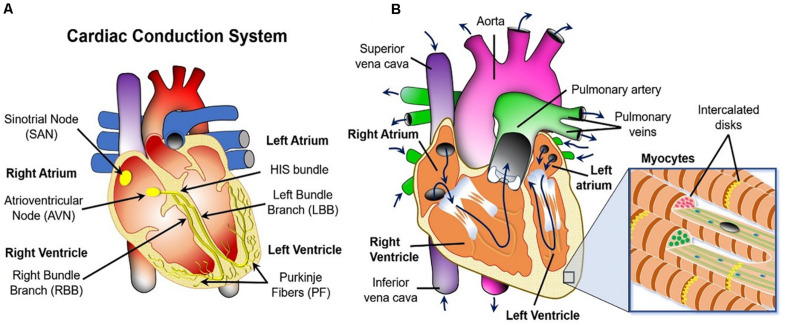
Macroscopic overview of the three physics occurring in the heart. **(A)** Cardiac conduction system schematic. **(B)** Blood flow path (navy blue arrows) and the fibrous structure defining the biomechanics of the heart wall (inset).

Figure 2A illustrates the cardiac conduction system (CCS) – a heterogeneous complex 3D network of highly specialized conductive cells (SA node, AV node, bundle of His, bundle branches, and Purkinje fibers) that transfer signals through the heart and cause it to contract. The electrical activity is initiated at the sinoatrial node (i.e., the natural pacemaker of the heart) where voltage signals called “action potentials” are produced periodically. Next the signals travel to the AV node, through the Bundle of HIS, down its branches and through the Purkinje Fibers. Ultimately, they are propagated to the myocardium (i.e., the muscular tissue of the heart) through discrete sites called Purkinje-Myocyte Junctions (not shown), causing the left and the right ventricles to contract independently of each other. This creates a double pumping action of the blood (see Figure 2B).

Specifically, oxygen-poor blood returns from the body to the right side of the heart (i.e., atrium and ventricle), which then sends it to the lungs for re-oxygenation. Oxygen-rich blood from the lungs then enters the left side of the heart and is pumped through the aorta back to the body. The blood can also carry thrombi (or their embolized pieces) from other parts of the body, and/or the clots could be generated within the heart itself via activation of platelets (blood cells responsible for clot formation) and the coagulation cascade (a series of biochemical reactions that results in the formation of a polymer mesh that facilitates the structural integrity of the clot). The presence of these objects in the cardiovascular system can interfere with the mechanical action of the heart by creating rigid obstructions. Furthermore, the blood clots can also get stuck in the cardio-vasculature and block the delivery of metabolites to the heart tissue. This leads to necrosis of the latter, commonly referred to as an ischemia or a heart attack.

### Structural Importance of the Myocardium

The organization of the cardiomyocyte fibers in the heart’s walls is thought to be critical to both the conductive and to the mechanical properties of the organ. Specifically, the contractile myocytes cells that cause the heart ventricles to beat are arranged in fibers (see inset of Figure 2B). These fibers make up the walls of the heart, which are lined with collagen and elastin extracellular matrix on the inside (i.e., endocardium) and the outside (i.e., epicardium). Their thickness varies both spatially and temporarily throughout the cardiac cycle.

**FIGURE 3:**
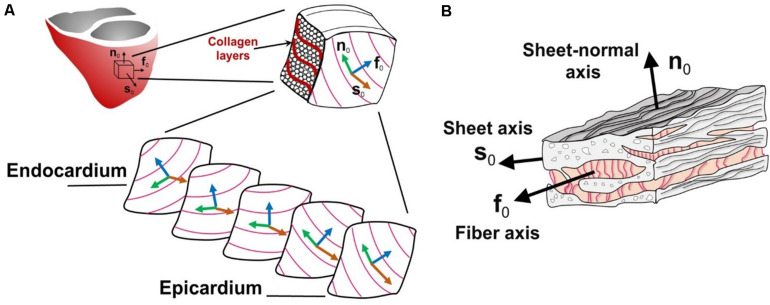
Schematic diagram of the heart tissue microstructure. **(A)** The transmural configuration of the muscle fibers and laminar sheets. **(B)** The layered organization of myocytes and the collagen fibers between the sheets. f_0_, fiber axis; s_0_, sheet axis and n_0_, sheet-normal axis. (Adopted with permission from Holzapfel and Ogden, 2009).

If one were to take a representative sample from the left ventricle (which pumps the most blood) as in Figure 3A, it would be possible to see that the 3D layered organization of the myocytes changes throughout the wall thickness from the epicardium to the endocardium. In fact, Figure 3A shows that the muscle fiber direction rotates from +50° to +70° (sub-epicardial region) to nearly 0° in the mid-wall region to −50° to −70° (sub-endocardial region) with respect to the circumferential direction of the left ventricle (Holzapfel and Ogden, 2009). Finally, Figure 3B show that the myocyte fibers (or myofibrils) are arranged into composite layers (or sheets), which are interconnected by collagen fibers. Therefore, for IBM to be physiologically representative, it must account for how this intricate structure affects the complex physics that occur in the heart.

## Geometry Module

The most common ways for obtaining a realistic macroscopic morphology of the heart and its surrounding blood vessels are computerized tomography (CT) and magnetic resonance imaging (MRI). However, the typical MRI/CT machines provide relatively coarse resolution datasets of the personalized cardiac geometry, with large gaps between slices (Schulte et al., 2001; Frangi et al., 2002; Appleton et al., 2005). This necessitates the use of interpolation procedures. Hence, the microscopic details (e.g., blood vessels, trabeculations, the Purkinje Fibers, the location and activity of the PMJs, the orientations of the myofibril sheets, etc.) are harder to resolve due to their small size. Yet, they strongly determine the electrophysiological and biomechanical properties of cardiac tissue (Watson et al., 2018). Consequently, there are three main methods for accounting for these fine details:

### Rule-Based Heuristics

**FIGURE 4:**
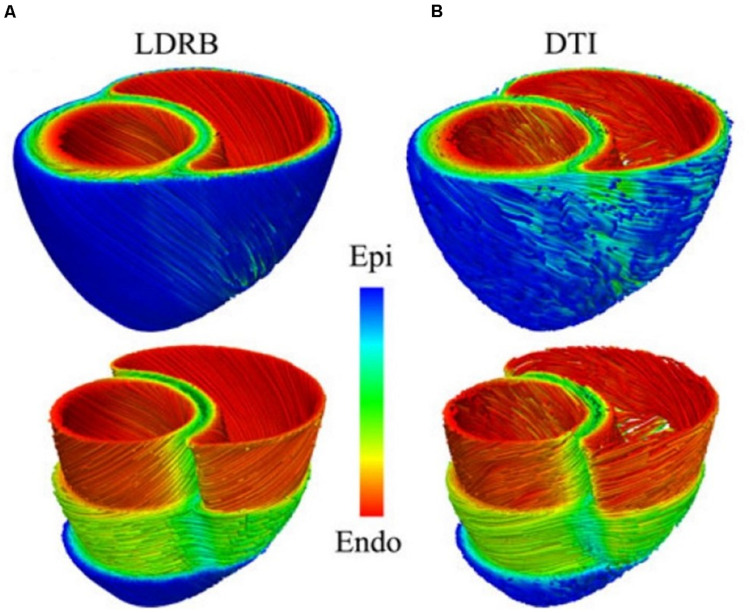
Comparison between **(A)** rule-based method and **(B)** DTI-based estimation of the myocardial fiber orientation for a 3D model of canine ventricles. (Reproduced with permission from Bayer et al., 2012).

The most rudimentary approach is to generate these features mathematically, based on observed trends (see Figure 4A) (Streeter Daniel et al., 1969). Briefly, the longitudinal fiber direction is assumed to rotate clockwise from the endocardium to the epicardium. Specifically, it is made parallel to the long axis of the papillary muscles, trabeculae at these regions and parallel to the endocardial and epicardial surfaces at the ventricular walls. Lastly, the fiber orientation in the septum is assumed to be running along the ventricular walls (Bayer et al., 2012). A popular way to personalize the algorithm to a patient specific structure of the heart is to use the minimal distance between the *imaged* endocardial and the epicardial surfaces to approximate orientation of the fibers. More stable and advanced rule based approaches, such as the Laplace-Dirichlet method also exist (Bayer et al., 2012). However, the heuristics are not guaranteed to be physiologically accurate, nor are they fully patient specific. Yet they remain the most common approach due to their low cost and ease of implementation, as well as due to the difficulty of imaging the microstructural details in a beating heart *in vivo*. And, as Figure 4 shows, they yield results that are comparable with the best of the imaging techniques (which typically require for the heart to be explanted and fixed in order to acquire such fine details).

### Histology/Optical Microscopy

The fiber orientation can also be approximated from histology of explanted hearts (Vetter and McCulloch, 1998; Deng et al., 2012), where the tissue is sliced into very thin 2D sections and dyed using special agents that highlight the features of interest. Although it is possible to create a 3D reconstruction based on the 2D slices using this approach, manual sectioning of the tissue may result in uneven slice thicknesses and feature distortion (Burton et al., 2006). Additionally, confocal microscopy has been used to image the fine structure of the myocardium (Hooks et al., 2002, 2006). However, the light penetration depth into the sample is typically limited to ∼100 mm. Hence, imaging a whole heart using this technique is also impractical. Therefore, both histology and confocal are typically used to provide localized information on explanted samples only. However, this information is useful for validating the rule-based methods, the *in vivo* imaging, and the modeling results.

### Diffusion-Tensor MRI, Micro-CT With Contrast and 3D Ultrasound Backscatter Tensor Imaging

Additional detail can be obtained from MRI images using a Gadolinium contrast agent (Bishop et al., 2010) and a special technique called Diffusion Tensor imaging (DTI) (see Figure 4B). The latter maps the diffusion of water molecules in the biological tissues, which is not free, but reflects the interactions with obstacles like macromolecules, fibers and membranes. For the cardiac DTI, it is well known that the direction of the primary eigenvector corresponding to each voxel of the received images matches the longitudinal axis of cardiac myocytes (Scollan et al., 1998; Holmes et al., 2000). This information can then be mapped onto the volumetric mesh of a 3D cardiac macro-geometry to include the microscopic fiber orientation (Plank et al., 2009; Vadakkumpadan et al., 2010). Likewise, micro-computed tomography (mCT) with iodine staining has also recently been used to assess the myocyte fiber orientation in the heart tissue (Aslanidi et al., 2013). However, both techniques are too slow to capture a beating heart in 3D without motion artifacts. Luckily, advanced ultrasound-based imaging techniques are coming online, which can map the myocardial fibers orientation and its dynamics with a temporal resolution of 10 ms during a single cardiac cycle, non-invasively and *in-vivo* in entire volumes (Papadacci et al., 2017). However, given the novelty, complexity and cost of these techniques, they are not yet widely available to the majority of the cardiovascular IBM researchers.

### Imaging-to-Modeling Pipeline

Perhaps the most difficult aspect of the *in vivo* scanning of live hearts is the need to perform significant image alignment using “registration” techniques. Furthermore, once the images are aligned, they must be “segmented” to identify the various tissue types and structural features of interest within the data. The segmentation can be either based on contrast dyes and/or on morphological feature detection (both manual and automated). The images can also be enhanced using digital post-processing, such as deconvolution (i.e., minimizing noise caused by objects outside of the imaging plane) and structure tensor analysis (e.g., enhancing visibility of the structural features for the fiber orientation detection) (Burton et al., 2006; Zhao et al., 2013). Numerical interpolation and machine learning techniques can further enhance the apparent resolution of the images digitally. Finally, the cardiovascular geometry must be “meshed” (i.e., broken up into pieces) to discretize the objects obtained from the images as a set of finite elements for numerical analysis. The latter is a simulation necessity that enables solving systems of complex (e.g., partial differential) equations by recasting them as *algebraic* approximations of the true solution. The trade-off for the simplified math is that the solution accuracy must be increased by making the mesh finer. This grows the number of equations that must be solved simultaneously, and thus the computational resource and time requirements. Overall, the imaging pipeline procedures are often very complicated and necessitate manual labor. This is both cumbersome (e.g., due to the large size of the high-resolution images) and subjective (e.g., due to the lack of contrast agents which necessitate user input). Therefore, there is an on-going effort to automate the imaging-to-modeling pipeline (Bishop et al., 2010).

## Electrophysiology Module

The simplest types of the heart models tend to be focused on cardiac arrhythmias. This is an “umbrella” term for irregularities in the conduction or pacing of the electrical signals that control the heartbeat rhythm. Given that some arrhythmias can lead to mortality, it is important to understand the underlying electrophysiological mechanisms of these disorders. Yet, electrocardiograms of the heart provide only limited information, which often fails to predict lethal outcomes (Goldberger et al., 2011). Therefore, computational modeling offers a better alternative for studying these diseases.

**FIGURE 5:**
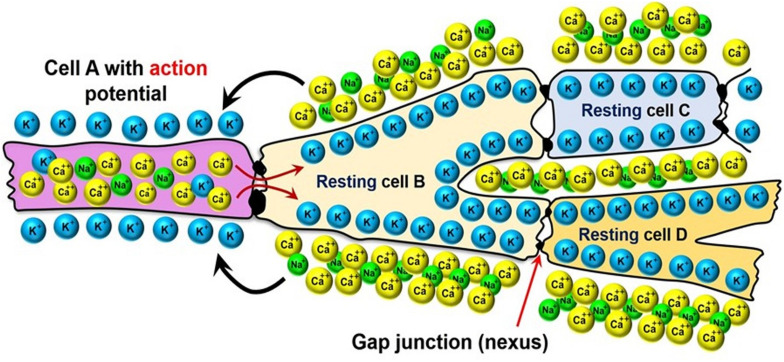
Electrical coupling of the neighboring cardiomyocytes via the gap-junctions between their membranes.

Most arrhythmia models focus on the electrophysiology of the heart, while assuming that it is isolated from the biomechanics of the contractions that lead to the pumping of the blood. As mentioned in Section “Methods: Literature Search,” the contraction of cardiomyocytes is initiated by electrical impulses called “action potentials,” which travel through the cardiac conduction system (see Figure 2A) into the myocardium. This electrical potential travels from one cell to another in the form of ions that pass through gap junctions between the cardiomyocytes (see Figure 5).

The cardiomyocytes are polarized, meaning that there is an electrical potential across the cell membrane: in the resting state the cells are more negative on the inside and positive on the outside, while the charge polarity is temporarily reversed as the action potential passes through them. This reversal occurs via the transport of Ca^++^ and Na^+^ ions from the outside of the cells to their inside, and K^+^ ions in the reverse direction (see Figure 5). The internalization of the Ca^++^ ion is especially important to the contraction of the cardiomyocytes, because it triggers a sub-cellular signaling cascade that generates tension inside of the cells. Therefore, the electrophysiology models simulate the propagation of the ionic currents through the myocardium.

**FIGURE 6:**
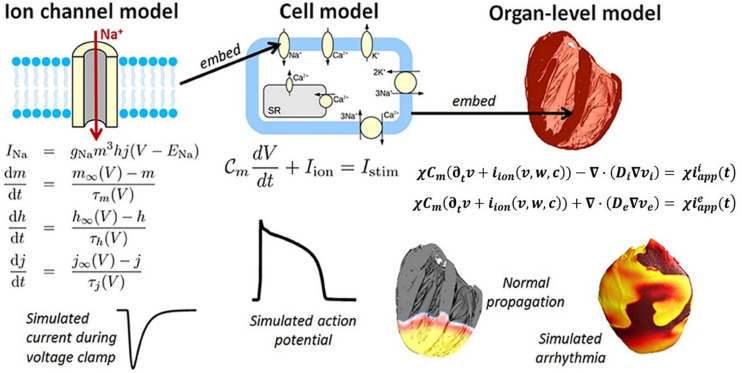
Components of a multiscale cardiac electrophysiology model. **(Left)** Equations and sample output for a Hodgkin-Huxley formulation of the rapid sodium current through an ion channel. Multiple such sub-cellular models can be used to define a cell model. **(Center)** Schematic of sub-cellular processes included in a hypothetical cell model, together with the differential equation governing the transmembrane voltage, and sample output. Cell models differ in their formulation of the ionic current *i*_*ion*_ and can be made up of dozens of ordinary differential equations. **(Right)** Cell models can be incorporated into the bidomain equations and solved on a computational mesh of the heart (**top right:** high-resolution rabbit biventricular mesh of Bishop et al., 2010), to simulate normal or arrhythmic cardiac activity **(bottom right)**. (Adopted with permission from Pathmanathan and Gray, 2018).

However, given that there are many cells in the heart, and each one of them has the ionic channels and transmembrane potentials, the problem is an inherently multiscale one (see Figure 6). On the subcellular scale (see Figure 6-LEFT), differential equations are used to model the transport of ionic species based on the Hodgkin–Huxley formulation (originally developed for the propagation of action potentials in neurons) (Hodgkin and Huxley, 1952). The subcellular models are then combined into cell scale models that can account for up to dozens of different ionic species and signaling intra-cellular cascades (see Figure 6, CENTER). Among these, the leading model is currently considered to be by O’Hara et al. (2011), which is based on experimental data from >150 undiseased human hearts. Finally, the individual ion currents are used to calculate the overall transmembrane potential, and its transport across the myocardium is treated as a diffusion across a homogenous medium (i.e., no discrete cells are considered by these models).

There are two types of formulations for the organ-level diffusion (which is related to the ionic conductivity) of the action potential across the myocardium: (1) the *bidomain* formulation, which considers different diffusivities inside and outside of the cell (Quarteroni et al., 2017) (see Figure 6, RIGHT):

(1) $$\begin{array}{c} \chi \,C_{m}\,\left(\partial_{t}v+i_{i\,o\,n}\,\left(v,w,c\right)\right)-\nabla \cdot \left(D_{i}\,\nabla v_{i}\right)=\chi \,i_{a\,p\,p}^{i}\,\left(t\right)\\ \chi \,C_{m}\,\left(\partial_{t}v+i_{i\,o\,n}\,\left(v,w,c\right)\right)-\nabla \cdot \left(D_{e}\,\nabla v_{e}\right)=\chi \,i_{a\,p\,p}^{e}\,\left(t\right)\\ \frac{d\,w}{d\,t}=m_{w}\,\left(v,w,c\right)\\ \frac{d\,c}{d\,t}=m_{c}\,\left(v,w,c\right) \end{array}$$

The bidomain formulation is used for simulating the action potential propagation throughout the myocardium in the intra- and the extra- domains separately. The myocardium is assumed to be a continuum in which the potential is considered to vary along the longitudinal direction of the conducting cells, while it is constant in the transversal (or radial) directions (Quarteroni et al., 2017). In this formulation, *v*_*i*_, *v*_*e*_ and ‘*v*’ are intracellular, extracellular and transmembrane potentials, respectively; $i_{a\,p\,p}^{i}$ and $i_{a\,p\,p}^{e}$stand for applied stimuli on the intra- and extracellular spaces, respectively; *i*_*ion*_ are the ionic currents following a Hodgkin–Huxley-type description for different ionic species (Hodgkin and Huxley, 1952); *w* are gating variables taking values in [0,1] that regulate the transmembrane currents and have a mutual relation with the intracellular concentrations *c* of different ionic species (which also vary depending on the values of transmembrane potentials *v*) (Quarteroni et al., 2017); *C*_*m*_ is the membrane capacitance; ‘χ’ is the ratio of membrane area per tissue volume; *D*_*i*_ and *D*_*e*_ are the conductivity tensors of the intra- and extracellular media, respectively.

and (2) the *monodomain* formulation, which simplifies the problem by considering only the transmembrane potential (Quarteroni et al., 2017):

(2) $$\chi \,\left[C_{m}\,\partial_{t}v+i_{i\,o\,n}\,\left(v,w,c\right)-i_{a\,p\,p}\,\left(t\right)\right]=\frac{1}{J}\,\nabla \cdot \left(D_{0}\,\nabla_{v}\right)$$

In this formulation, the cardiac tissue is also assumed to be a continuum, but the current conservation is written in terms of the transmembrane potential *v* only (i.e., not considering the intra- and extracellular potentials) (Quarteroni et al., 2017). Instead, the intracellular and extracellular diffusivities are assumed to be proportional to each other, and therefore can be represented by a single variable. Herein, *D*_0_ is the conductivity tensor in a fixed reference state, *J* is the determinant of the deformation gradient tensor, which represents the volume change of a deformable object. The trade-off for the simplicity is that the monodomain model is unable to describe cardiomyocyte repolarization patterns. For this reason, the bidomain model is more widely used (Bishop and Plank, 2011).

### Module Personalization

**FIGURE 7:**
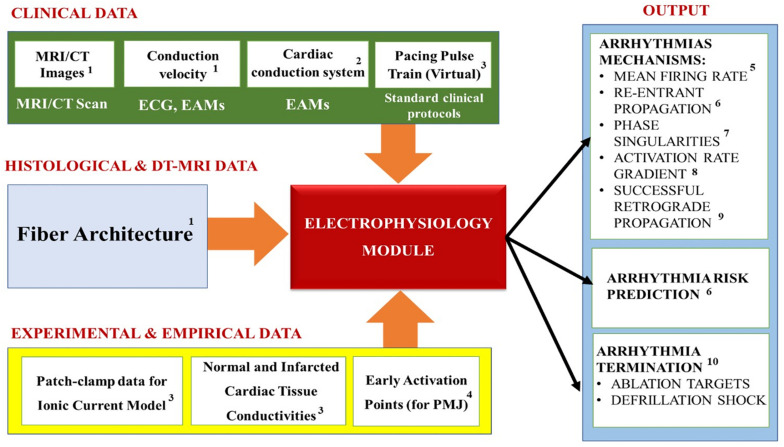
Summary of the Electrophysiology module’s inputs, outputs and applications. Superscripts in the figure correspond to the following references: ^1^Cardenes et al., 2014; Kayvanpour et al., 2015; Lopez-Perez et al., 2015; ^2^Cardenes et al., 2014; Palamara et al., 2014; ^3^Arevalo et al., 2016; Trayanova et al., 2017; ^4^Lopez-Perez et al., 2015; ^5^Behradfar et al., 2014; ^6^Arevalo et al., 2016; Deng et al., 2016; Trayanova and Chang, 2016; Prakosa et al., 2018; ^7^Boyle et al., 2016; Roney et al., 2018; ^8^Smith et al., 2011; Boyle et al., 2013; Potse et al., 2014; ^9^Trayanova, 2011; Behradfar et al., 2014; ^10^Arevalo et al., 2016; Trayanova et al., 2017; Deng et al., 2019.

Table 1 summarizes the most common electrophysiology module personalization approaches encountered in the recent IBM works, while Figure 7 maps the relationships between the module’s inputs, outputs and applications. In this, and in the subsequent modules, the heart’s *macroscopic* anatomy can be personalized for a specific patient by acquiring its geometry from the *in vivo* imaging (see “Geometry Module” section). Furthermore, pathological tissue remodeling (e.g., locations and extension of infarct scars, diffuse fibrosis, etc.) can be accounted for via the imaging as well (Vadakkumpadan et al., 2009; Mewton et al., 2011; Dass et al., 2012; Ashikaga et al., 2013; Arevalo et al., 2016; Trayanova et al., 2017). However, there are two important types of *microscopic* structural information that cannot be completely personalized yet: the myocardial fiber orientation and the CCS.

**TABLE 1:** A recent literature survey of how the Electrophysiology Module is typically personalized using image-based information.

**Imaging method**	**Personalized information from imaging**	**Mapping of the personalized information from imaging to the module’s inputs**	**Citation**
MRI	Geometry with infarcted regions	Geometry was personalized and tissue conductivity values were adjusted to match the conduction velocity (CV) in human cardiac tissue.	Ashikaga et al., 2013
MRI	Geometry with infarcted regions	Geometry was personalized and tissue conductivity values were adjusted to match the CV in human cardiac tissue.	Arevalo et al., 2016
MRI	Geometry with infarcted regions	Geometry was personalized and tissue conductivity values were adjusted to match the CV in human cardiac tissue.	Trayanova et al., 2017
CT	Geometry	Geometry was personalized and tissue conductivities were adjusted to fit local activation times, which were obtained using electro-anatomical maps (EAMs).	Cardenes et al., 2014
CT and MRI	Geometry with infarcted regions	Geometry was personalized and tissue conductivities were adjusted to fit patient-specific electrical activation using EAMs.	Palamara et al., 2014
MRI	Geometry	Geometry was personalized; tissue conductivities were adjusted to fit patient-specific electrical activation and ECG; and ionic currents were also personalized (via blood electrolyte concentrations measurement).	Krueger et al., 2013a
MRI	Geometry with infarcted regions	Geometry was personalized; tissue conductivities were adjusted to fit a human cardiac tissue model; and ionic currents were personalized via blood electrolyte concentrations measurements.	Krueger et al., 2013b
Cardiac delayed enhancement-MRI	Geometry with infarcted regions, papillary muscles and main endocardial trabeculations	Geometry was personalized and tissue conductivity values were adjusted to match the CV in human cardiac tissue.	Lopez-Perez et al., 2019
MRI	Geometry	Geometry was personalized and tissue conductivities were adjusted to match clinically measured propagation times of the patient using EAMs.	Sermesant et al., 2008
MRI	Geometry with infarcted regions	Geometry was personalized and tissue conductivity values were adjusted to match the CV in human cardiac tissue.	Bruegmann et al., 2016
Cine MRI (with torso geometries)	Geometry with orientation and position of heart	Geometry was personalized and tissue conductivity values were adjusted to match patient-specific ECG data.	Kayvanpour et al., 2015
MRI (with torso geometries)	Geometry with orientation and position of heart	Geometry was personalized and tissue conductivity values were adjusted to match the CV in human cardiac tissue.	Mincholé et al., 2019
MRI	Geometry with infarcted regions	Geometry was personalized and tissue conductivity values were adjusted to match the CV in human cardiac tissue.	Deng et al., 2016
MRI	Geometry with infarcted regions	Geometry was personalized and tissue conductivity values were adjusted to match the CV in human cardiac tissue.	Prakosa et al., 2018
Late gadolinium enhancement MRI	Detailed geometry, including the atrial structure, mitral and tricuspid valves, coronary sinus, pulmonary veins, superior and inferior vena cava.	Geometry was personalized and tissue conductivity values were adjusted to match patient-specific activation mapping data using EAMs.	Roney et al., 2018
Cardiac magnetic resonance procedure	Detailed geometry, including the atrial structure, aortic arch, caval veins, torso surface, trabeculated myocardium between the wall and the intracavitary blood.	Geometry was personalized and tissue conductivity values were tuned to match patient-specific activation mapping using EAMs and ECG data.	Potse et al., 2014

Both are, unfortunately, still too difficult to image in a moving heart *in vivo*. Thus, typically an *approximated* personalization is performed to include these microscopic features using the rule-based algorithms (see “Geometry Module” section). Additional CCS approximation methods are based on: early activation points obtained from the literature (Durrer et al., 1970); manual delineation of CCS on the endocardial surfaces (Romero et al., 2010); *ex vivo* data obtained by means of histological studies of animal hearts (Sebastian et al., 2013); from *in vivo* electro-anatomical maps (EAMs) (Cardenes et al., 2014; Palamara et al., 2014). The EAMs data can provide the location of some of the PMJs, and can also be used to reconstruct patient-specific electrical activation patterns (Cardenes et al., 2014; Palamara et al., 2014).

Likewise, the conductivity values of different tissue zones (normal and abnormal/damaged) are typically too difficult to measure in living human patients, and are thus initialized based on accepted literature values: 2–3 m/s in the His-Purkinje system and 0.3–0.4 m/s in the conductive myocardial cells (Ideker et al., 2009). They are then either further adjusted to match the human myocardium conduction velocity (CV) (i.e., speed at which action potentials are distributed throughout the tissue) measured using explanted hearts (Arevalo et al., 2016; Trayanova et al., 2017; Lopez-Perez et al., 2019), or are tuned to fit patient-specific electrical activation patterns obtained from electrocardiograms (ECG), body surface potential maps (BSPM) or EAM (Sermesant et al., 2008; Lopez-Perez et al., 2015).

Unfortunately, at the cellular level, the patient-specific transmembrane current dynamics (i.e., *i*_*ion*_) cannot yet be measured; and hence, the existing mathematical models are not personalized at such detail. Similarly, the electrical heterogeneity between the different regions (e.g., transmural heterogeneity in the ventricular walls), the electrical remodeling and the effects due to an individual’s genetic mutations on the cardiac electrophysiology cannot yet be accounted for (Lopez-Perez et al., 2015). However, a cellular level electrophysiology model that best matches the patient’s pathology can instead be chosen from either existing literature datasets that are representative of a patient-group (Krueger et al., 2013a,b) or from patch-clamp studies of cells harvested from pathologic zones of the patient (Cabo and Boyden, 2003; Decker and Rudy, 2010). Additionally, the extracellular ion concentrations can be estimated and set into a model from personalized measurements of blood electrolyte concentrations (Krueger et al., 2013a,b).

### Module Outputs and Applications

Overall, the electrophysiology modeling studies the normal conduction in the heart, as well as the pathological mechanisms that arise and cause cardiac arrhythmias. It is typically used to calculate physiological parameters (see Figure 7) like: the Mean firing rate (i.e., the number of spikes during a cardiac cycle divided by cycle duration, spike/s) (Behradfar et al., 2014); Re-entrant arrhythmias propagation (Arevalo et al., 2016; Deng et al., 2016; Trayanova and Chang, 2016; Prakosa et al., 2018) (i.e., a propagation of an impulse that fails to die out after normal activation of the heart and continues to re-excite it after the refractory period has ended, Antzelevitch, 2001); Phase singularities (Boyle et al., 2016; Pathmanathan and Gray, 2018; Roney et al., 2018) which represent the sites in which the activation state cannot be determined, because the particular location is surrounded by activation states ranging from fully activated to fully recovered (Valderrabano et al., 2003); Activation rate gradient which quantifies how fast the transmembrane voltage *V*_*m*_ changes in different cardiac regions (Smith et al., 2011; Boyle et al., 2013; Potse et al., 2014); Successful retrograde propagation which measures whether conduction at a terminal Purkinje node is successful or refractory (Trayanova, 2011; Behradfar et al., 2014); and the organization of electrical wavelets as they propagate through the myocardium (Starobin et al., 1996; Keldermann et al., 2009; Trayanova, 2014). In our experience, the majority of the electrophysiological modeling is used for elucidating the mechanisms of cardiac arrhythmia, especially for “reentrant propagation of complex waves” (e.g., effects of cardiac microstructure, spiral wave breakup, early afterdepolarizations, scroll-wave filaments, action potential duration, electrical alternans, etc.) (Trayanova, 2011); as well as for prediction of arrhythmia risks in specific patients. Furthermore, these models are also used to examine the mechanisms of defibrillation shock in the heart for terminating arrhythmia, as well as for increasing the understanding of ablation targets in the arrhythmia treatments (Trayanova et al., 2017).

### Module Personalization Example

The following is a discussion of a representative example of the personalized electrophysiology modeling applied to an arrythmia risk assessment in post-infarcted hearts. Specifically, personalized 3D computer models of the post-infarction hearts was constructed based on clinical MRI of specific patients. First, an individualized geometric model of the postinfarction ventricles was reconstructed from late-gadolinium-enhanced-MRI (Arevalo et al., 2016), with representations of both the scar and the infarcted border zones. Due to the difficulty imaging the myocardial fiber orientation from a moving heart *in vivo*, an approximated personalization was performed using a rule-based algorithm (Bayer et al., 2012). Region-specific cell and tissue electrical properties were then assigned to the electrophysiological model based on literature data. After that, a virtual multi-site delivery of electrical stimuli from various bi-ventricular locations was conducted, in order to computationally determine all the ventricular tachycardia reentrant pathways that the infarct-remodeled ventricular substrate can sustain. This methodology was then validated in an arrhythmia risk prediction clinical study including 41 patients and significantly surpassed several existing clinical metrics in predicting upcoming arrhythmic events (Arevalo et al., 2016).

## Biomechanics Module

**FIGURE 8:**
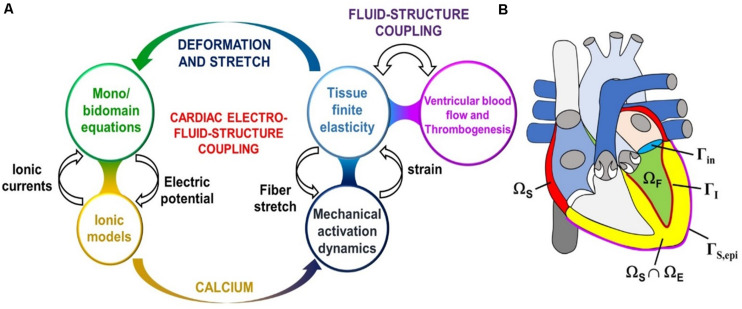
**(A)** Sketch of the cardiac electro-fluid–structure coupling. **(B)** The three computational domains (fluid domain Ω_F_, solid mechanics domain Ω_S_, electrophysiology domain Ω_E_) considered in the cardiac multiphysics problem. In this example the electrophysiology physics are only considered in the ventricles, whereas the solid mechanics physics applies to the atria also. Additionally pictured are the fluid–structure interface Γ_I_ inside the left ventricle, the epicardial surface Γ_S,epi_, and the mitral valve inlet surface Γ_in_. Lastly, the domains Ω_E_ and Ω_S_ overlap within the ventricular part of the heart. (Adopted with permission from Quarteroni et al., 2017).

The next level of complexity are the models of myocardial abnormalities/heart failure (HF) and the blood pumping assist devices such as the Left-Ventricular Assist Device (LVAD). Since these models are interested in how the presence of abnormalities or assist devices affects the blood circulation, they must account for the contraction solid mechanics and the blood hemodynamics. However, if they are not interested in clot formation, the models are simplified by homogenizing the blood flow. Therefore, they are not as complicated as the ones that do account for the thrombosis. Yet, they are still complex, because they include solving multiple different types of coupled physics (see Figure 8, LEFT) applied in different parts of the heart (see Figure 8, RIGHT).

**FIGURE 9:**
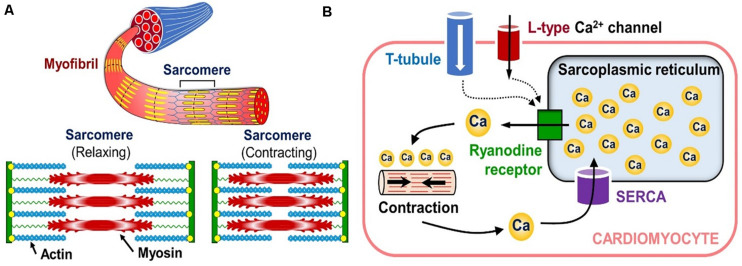
Sub-cellular tension generation mechanisms. **(A)** Organization of the cardiac muscle cell’s contraction mechanism (bottom inset shows the contraction-relaxation cycle of a single sarcomere). **(B)** Diagram showing how calcium release from internal cytosol storage causes cardiomyocyte contraction in response to the “outside-to-inside” calcium signaling.

Since the electrophysiology in these models is treated similarly to the arrhythmia models, the cardio biomechanics framework will be discussed next. Central to this physics type is the fact that the myocytes in the heart contain rod-like structures called myofibrils, which are composed of repeating contractile units called “sarcomeres” (see Figure 9, LEFT). Each sarcomere contains thin and thick filaments, made from actin and myosin proteins, respectively.

After depolarization, calcium enters the cardio-myocytes through the ion channels in their membrane, and triggers the release of cytosolic calcium stored in the sarcoplasmic reticulum (a storage compartment) of the cells through a cascade of intra-cellular signaling (see Figure 9, RIGHT). This release of the internal calcium leads to the binding of myosin heads to the actin filaments in the sarcomeres, which in turn causes the filaments to slide against each other and contract the entire cell (see bottom inset in Figure 9, LEFT). This process is called the “crossbridge mechanism” and it corresponds to the *active* tension in the biomechanical calculations of the heart.

The kinetics of this process have been modeled using Monte Carlo (Walcott and Sun, 2009) and partial differential equations (Huxley, 1957). However, both approaches are too computationally expensive to be calculated at the organ level. Consequently, the latter model has been simplified by considering a single cross bridge representative of the whole distribution (i.e., mean field theory) (Negroni and Lascano, 2008; Rice et al., 2008; Washio et al., 2012) or by averaging the distributions over a single cell (Bestel et al., 2001). Simpler yet is the assumption that the active contraction of the individual cells depends on the intracellular ionic concentrations and the local deformation gradient (Quarteroni et al., 2017). Ultimately, the microscopic sarcomere sliding velocity is related to the macroscopic strain along the myocardial fibers via a constitutive relationship (i.e., microscopic rate-of-strain depends on the macroscopic strain), such as the Hill-Maxwell rheological model which is a modification from Hill’s force-velocity relation (Sainte-Marie et al., 2006). Herein, a specific choice of the attachment and detachment rates was modified so that they were not only dependent upon the sarcomere strain, but also on the strain rate.

The active contraction can be expressed by the following active stress formulation from Rossi et al. (2014):

(3) $$P_{A}=\left(\frac{\partial W_{A}}{\partial I_{1}^{E}}+\frac{\partial W_{A}}{\partial I_{4,f}^{E}}\right)\,{\hat{F}}_{A}\,\left(c,I_{4,f}\right)\,f_{0}⊗f_{0}+\frac{\partial W_{A}}{\partial F_{A}}$$

And the active strain formulation can be derived from Quarteroni et al. (2017):

$$\partial_{t}\gamma_{f}=\frac{1}{\eta_{A}}[\left(\frac{\partial W_{A}}{\partial I_{1}^{E}}+\frac{\partial W_{A}}{\partial I_{4,f}^{E}}\right)\left({\hat{F}}_{A}\left(c,I_{4,f}\right)-\frac{2\,I_{4,f}}{{\left(1+\gamma_{f}\right)}^{3}}\right)$$

(4) $$\;-\frac{\partial W_{A}}{\partial F_{A}}:f_{0}⊗f_{0}]$$

Where, *W*_*A*_ is the *active* component of the free energy, *F*_*A*_ is the active deformation, ‘*c’* is the intracellular calcium concentration, *I*_4,f_ is the local deformation gradient invariant in the myofiber direction, $\partial I_{1}^{E}$ and $\partial I_{4,f}^{E}$ are elastic invariants (described in more details in Rossi et al., 2014). The incorporation of the microscopic active tension, ${\hat{F}}_{A}$, at the tissue level is a key aspect in the multi-scale framework of the cardiac function (Quarteroni et al., 2017) and can be derived by:

(5) $${\hat{F}}_{A}\,\left(c,I_{4,f}\right)=\alpha \,f\,\left(c\right)\,R_{F-L}\,\left(I_{4,f}\right)$$

where *R*_*F*−*L*_(*I*_4,*f*_) is a function that represents the force–length relationship of the cardiac cells (defined in Ruiz-Baier et al., 2014) which depends on *I*_4,f_; *f* (*c*) specifies the amount of force generated by the cross-bridges in response to intracellular calcium release; and α is a positive parameter. Thus, the ${\hat{F}}_{A}$ represents the active tension generated within the sarcomeres, which then drives the macroscopic muscular contractions.

In addition to the active tension generated by the cross-bridge mechanism, the solid mechanics calculations must also account for the passive stiffness of the myocardium (e.g., when it is expanded by the blood flow entering the heart). This is modeled by assuming that the myocardium is an isotropic linear elastic tissue. The general formulation of passive stress (i.e., Cauchy stress) can be expressed by Avazmohammadi et al. (2019):

(6) $$\sigma =J^{-1}\,F\,S\,F^{T}$$

Where, the second Piola–Kirchhoff stress tensor ‘*S’* can be described in terms of the stored energy density function ‘*W’* through: *S* = 2∂*W*/∂*C*, and ‘*W’* can be derived based on Holzapfel–Ogden constitutive law which is divided into three parts - the isotropic isochoric part, the isotropic volumetric part, and the orthotropic part:

$$W\,\left(F\right)=\frac{a}{2\,b}\,\exp\left(b\,\left[\overset{-}{I}-3\right]\right)+\frac{\kappa }{4}\,\left[{\left(J-1\right)}^{2}+{\left(\ln J\right)}^{2}\right]$$

+∑i=f,Sai2⁢bi⁢[exp⁡(bi⁢<I4,i-4,i-1>2)-1]

(7) +af⁢s2⁢bf⁢s⁢[exp⁡(bf⁢s⁢I8,f⁢s2-2)-1]

where

(8) $$\begin{array}{c} J=\det\left(F\right),C=F^{T}\,F,I_{1}=t\,r\,\left(C\right),\bar{I_{1}}=J^{-2/3}\,I_{1},\\ I_{4,f}=C\,f_{0}\cdot f_{0},{\bar{I}}_{4,f}=J^{-2/3}\,I_{4,f},I_{4,s}=C\,s_{0}\cdot s_{0},I_{4,s}=J^{-2/3}\,I_{4,s},\\ I_{8,f\,s}=c\,f_{0}\cdot s_{0},{\bar{I}}_{8,f,s}=C\,f_{0}\cdot s_{0},{\bar{I}}_{8,f,s}=J^{-2/3}\,I_{8,f,s} \end{array}$$

and the material parameters *a*, *a*_*f*_, *a*_*s*_, *a*_*fs*_, *b*, *b*_*f*_, *b*_*s*_, *b*_*fs*_ are experimentally fitted (Quarteroni et al., 2017). The parameter ‘*κ’* is the bulk modulus that “penalizes” local volume changes to enforce the incompressibility of the tissue.

Furthermore, since stretching a cell changes the distance between the gap junctions and their neighbors, this leads to changes in the ion channels, and consequently, in the conductivity of the action potential from cell to cell. This electromechanics coupling is typically included by modifying the conductivity tensor in the original equation of the electrophysiological propagation (see Equation 2). Specifically, the fixed reference state conductivity tensor ‘*D_0_’* is replaced with the spatial configuration conductivity tensor ‘*D’*. Furthermore, an explicit dependence on the solid deformation tensor ‘*F’* is included into the conductivity tensor in order to account for the geometric feedback, due to deformation of the tissue structure (Quarteroni et al., 2017):

$$\chi \,\left[C_{m}\,\partial_{t}v+i_{i\,o\,n}\,\left(v,w,c\right)+i_{S\,A\,C}\,\left(v,F\right)-i_{a\,p\,p}\,\left(t\right)\right]$$

(9) $$\;=\frac{1}{J}\,\nabla \cdot \left(J\,F^{-1}\,D\,F^{-T}\,\nabla v\right)$$

Moreover, an additional inward ionic current, induced by the stretch-activated channels, also contributes to the depolarization. This is commonly modeled as follows:

(10) $$i_{S\,A\,C}\,\left(v,F\right)=g\,\left(\sqrt{I_{4,f}\,\left(F\right)}-1\right)\,\left(v-E\right)$$

Where, ‘*E’* and ‘*g*’ are the reversal potential and the maximal conductance of the channels.

Lastly, *in vivo* the heart is immersed in a pericardial fluid; and it is also loosely supported by a flexible double-layered pericardium membrane. Therefore, spring-like external support enforced by Robin-type boundary conditions (Moireau et al., 2012) are typically tuned to mimic the global motion of the modeled heart. An explicit solution of the pericardium–heart contact problem has been proposed as well (Fritz et al., 2014).

### Module Personalization

**FIGURE 10:**
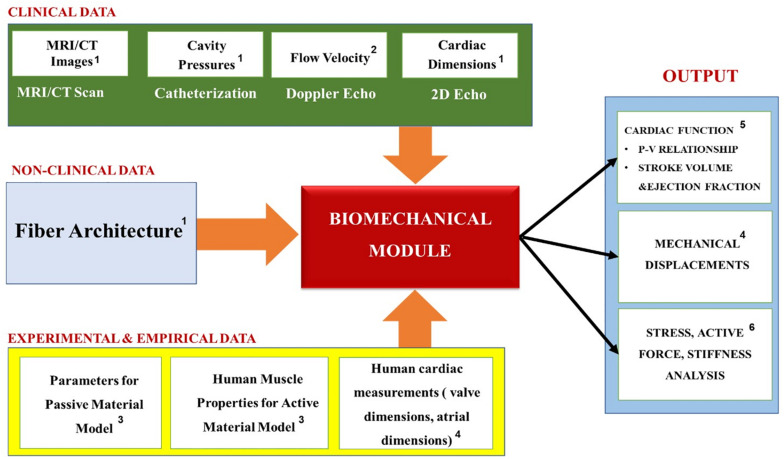
Summary of the Biomechanics module’s inputs, outputs and applications. Superscripts in the figure correspond to the following references: ^1^Krishnamurthy et al., 2013; Meoli et al., 2015; Finsberg et al., 2018; ^2^Krishnamurthy et al., 2013; Meoli et al., 2015; ^3^Krishnamurthy et al., 2013; Kayvanpour et al., 2015; Meoli et al., 2015; Finsberg et al., 2018; Palit et al., 2018; Shavik et al., 2020; ^4^Krishnamurthy et al., 2013; ^5^Krishnamurthy et al., 2013; Palit et al., 2018; ^6^Lee et al., 2013; Meoli et al., 2015; Finsberg et al., 2018.

Table 2 summarizes the most common biomechanics module personalization approaches encountered in the recent IBM publications, while Figure 10 the relationships between the module’s inputs, outputs and applications. Before personalizing the modeling of the passive (i.e., resting) properties of the myocardium, the parameters of the Holzapfel and Ogden model (see Equations 7 and 8) are typically initialized using experimental data from biaxial (Krishnamurthy et al., 2013) and shear tests (Dokos et al., 2002; Sommer et al., 2015) of *explanted* myocardial tissue. The passive mechanics parameters are then further optimized to match the patient-specific end-diastolic pressure and volume (EDPV) relations (Krishnamurthy et al., 2013; Meoli et al., 2015; Finsberg et al., 2018; Palit et al., 2018). The EDPV relation is a graphical representation of the pressure-volume loop related to the passive filling of the left ventricle during diastole (i.e., relaxation), and is a measure of the passive ventricle stiffness. The chamber volume of the left ventricle at the end of the diastole is defined as the end-diastolic volume (EDV), which is used to estimate the preloading volume of the heart and indicate the stiffness of the ventricle.

**TABLE 2:** A recent literature survey of how the Biomechanics module is typically personalized using image-based information.

**Imaging method**	**Personalized Information from Imaging**	**Mapping of the Personalized Information from the Imaging to the Module’s Inputs**	**Citation**
CT combined transthoracic 2D; and continuous-wave Doppler echocardiography	Geometry with Infarcted regions, ventricular dimensions including early diastolic volume, EDV, ESV and blood flow velocities	Geometry was personalized; passive mechanics parameters were fitted to match the previous experimental results on cardiac tissues and match patient-specific EDPV relations; active mechanics parameters were adjusted to match the patient-specific measured peak left ventricular pressures and ESV.	Krishnamurthy et al., 2013
DT-MRI from explanted heart	Fiber direction		
Cine cardiac MRI	Geometry with ventricular dimensions and ejection fraction (EF).	Geometry was personalized; passive mechanics parameters were fitted to match the previous experimental results and match the previous results of EDPV relations (due to the unavailability of subject-specific ventricular pressures that require invasive measurements).	Palit et al., 2018
Cine cardiac MRI	Geometry with ventricular dimensions and regional strain-time	Geometry was personalized; passive mechanics parameters were fitted to match patient-specific EDPV relations; active mechanics parameters were adjusted to match the patient-specific end-systolic state.	Finsberg et al., 2018
MRI flow tracing and echocardiographic Doppler velocity tracings	Geometry with ventricular dimensions and blood flow velocities.	Geometry was personalized; passive mechanics parameters were fitted to match the previous experimental results on cardiac tissues and match patient-specific EDPV relations; active mechanics parameters were adjusted to match the patient-specific measured peak left ventricular pressures.	Meoli et al., 2015
MRI	Geometry with ventricular dimensions	Geometry was personalized; passive parameters were tuned so that the resultant EDV matched the corresponding MRI-measured cavity volume; active parameters were adjusted so that the resultant ESV matched the corresponding MRI-measured cavity volume.	Lee et al., 2013
Cine MRI and Echocardiography	Geometry with ventricular dimensions and EF	Geometry was personalized; passive and active parameters were determined by minimizing the sum of the squared differences between computed and measured EF, stroke volume, EDV, ESV, end-diastolic pressure and end-systolic pressure.	Kayvanpour et al., 2015
Cine MRI	Geometry with ventricular volume waveforms	Geometry was personalized; biomechanics parameters were tuned along with hemodynamics parameters; passive parameters were adjusted to match the measured EDPV; active parameters were adjusted to match patient-specific volume and pressure waveforms.	Shavik et al., 2020
Phase-contrast MRI	Luminal area waveforms		

The active contraction modeling, on the other hand, is coupled with the cardiac hemodynamics, so it is important to link it with the patient-specific hemodynamic metrics corresponding with the end-systolic condition. For example, the pumping ability of the heart is represented by the end-systolic pressure (ESP) and the end-systolic volume (ESV), which are the peak values of pressure and ventricular volume at the end of systole (i.e., contraction), respectively. The active contraction is also associated with ejection fraction (EF), which is defined as a measurement of the percentage of blood leaving out of the left ventricle with each contraction. Therefore, the optimization procedure is performed to find the patient-specific parameters that best replicate the clinical hemodynamics of the patient in terms of the mean, maximum and minimum values of the pressures, flows and the cardiac volumes (such as the clinically recorded values of the ESP, ESV, and EF).

### Module Outputs and Applications

Overall, the biomechanics models tend to perform stress analysis, which is used to evaluate the effects that a defective myocardium structure has on the heart function, and design new therapies and treatment devices for reducing the abnormal stress (Guccione et al., 2003; Wall et al., 2006; Lee et al., 2013; Finsberg et al., 2018) (see Figure 10). Additionally, they calculate the stiffness of the heart, which strongly corresponds to its ability to function normally and can be used as an indicator for HF (e.g., heart attacks caused by a diastolic dysfunction that occurs when the ventricle becomes too stiff or weak to pump blood effectively). Ultimately, however, since the goal of these models is to obtain the relationship between the biomechanics of the myocardium tissue and the blood pumping ability of the heart, they also include a simplified (i.e., no discrete blood cells) hemodynamics module, which is discussed in the next section.

### Module Personalization Example

The following is a discussion of a representative example of the personalized Biomechanics module applied to five HF failure patients from San Diego Veteran’s Affairs Medical Center. Specifically, personalized 3D models of ventricular biomechanics in their failing hearts were derived from cardiac CT imaging. The human fiber orientation was modeled using DT-MRI data from an isolated (i.e., fixed) human organ-donor heart, and then transposed to the specific patient’s geometric model. The biomechanics model was then developed for optimizing the passive material properties to match previous experimental results on cardiac tissues and patient-specific end-diastolic pressure and volume relations. The material properties of the active contraction were also optimized to match patient-specific measured peak left ventricular pressures and end-systolic volumes. These components were then integrated to generate a multi-scale computational approach for the patient-specific hearts. The simulation results in the patients demonstrated good agreement with their measured echocardiographic and cardiac output parameters, such as EF and peak cavity pressures. This model was developed for stress analysis in HF patients and could be further developed with the goal of predicting treatments for heart disease under different interventions.

## Simplified Hemodynamics Module

In the cardiovascular models where clot formation is not considered, the blood flow is simulated using the incompressible Navier-Stokes equations. This means they do *not* account for discrete cells floating in the plasma. Instead the blood is treated as a *homogeneous* weakly non-Newtonian fluid (Shibeshi and Collins, 2005), which flows mostly in the laminar regime [though the strong vortices can create transition to turbulence with Reynolds numbers in the 1500–2500 range (Quarteroni et al., 2017)]. The fluid-structure interaction between the blood and the myocardium walls is typically modeled explicitly using moving mesh approaches: for example, Arbitrary Lagrangian-Eulerian (Cheng et al., 2005; Chnafa et al., 2014; Su et al., 2014), immersed boundary (Kohl et al., 2001; Vigmond et al., 2008) and level-set based methods (Mihalef et al., 2011). The heart valves, on the other hand, are commonly approximated using the Bernoulli equation for orifice flow (Flachskampf et al., 1990; Vandervoort Pieter et al., 1995; Donati et al., 2017).

**FIGURE 11:**
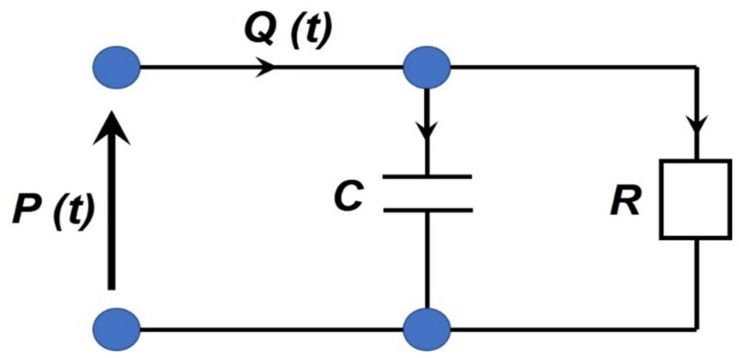
Two-element Windkessel circuit analogy illustrated.

Additionally, the myocardium hemodynamics are typically coupled to the rest of the body’s circulation via the Windkessel circuit model, which mimics the arterial blood pressure’s waveform. This is a relatively simple method used to obtain the relationship between the blood flow and the pressure in a modeled segment through the resistive ‘*R*’ and the capacitance ‘*C*’ properties of the arterial vasculature (see Figure 11) (Wall et al., 2006). Specifically, the heart and the systemic arterial system are treated as a closed hydraulic circuit, which, contains a pump connected to a chamber partially filled with a liquid. As it is pumped, the latter compresses the air pocket in the chamber, which in turn pushes the liquid back out (i.e., creating a back-and-forth cycle). Consequently, the arterial compliance, the peripheral resistance, and the inertia are modeled as a capacitor, a resistor, and an inductor, respectively. In this model, the physiological variables such as pressure ‘*P*’ and flow ‘*Q*’ only vary as a function of time ‘*t*’ (Morris et al., 2016). The relationship between the flow rate *Q(t)* and the pressure *P(t)* can be expressed by:

(11) $$Q\,\left(t\right)=\frac{P\,\left(t\right)}{R}+C\,\frac{d\,P\,\left(t\right)}{d\,t}$$

It essentially states that the volumetric flowrate must equal to the sum of the volume stored in the capacitive element and the volumetric outflow through the resistive element. During the diastole there is no blood inflow (*Q* = 0), so the Windkessel equation can be solved for the *P(t)*:

(12) $$P\,\left(t\right)=P\,\left(t_{d}\right)\,e^{\frac{-\left(t-t_{d}\right)}{\left(R\,C\right)}}$$

where *t*_*d*_ is the time of the start of the diastole and *P(t_*d*_)* is the blood pressure at that time. Due to its simplicity, the Windkessel equation is frequently used to approximate various components and boundary conditions in the cardiovascular system (Morris et al., 2016). However, this model is only a rough approximation of the arterial circulation. To that end, the next section covers the approaches to more detailed hemodynamic formulations.

## Hemodynamics With Thrombogenesis Module

The last class of the cardiovascular models is the one in which the hydrodynamics of the discrete blood cells, and the biochemistry associated with their physiological activity, are of interest. For example, such simulations could be focused on studying pathological clot formation inside of the heart (Choi et al., 2015; Mittal et al., 2016; Seo et al., 2016; Harfi et al., 2017), or embolism into it from other parts of the body. Therefore, these types of models must simulate the blood as a suspension of deformable cells (e.g., platelets and/or red blood cells), whose fate is intertwined with the mechanical motion of the myocardium (and by association with the electrophysiology of the heart). This is typically done using Stokesian Dynamics methods, Dissipative Particle Dynamics, Completed Double Layer Boundary Integration Equation Method and Lattice Boltzmann Method (Wang and King, 2012). These are mesoscopic off- and on- lattice frameworks that calculate trajectories of the cells under the influence of hydrodynamic and Brownian forces; while the deformation of the structure is typically simulated using continuum-based models that treat the cell membrane and intracellular fluids as homogeneous materials: some popular approaches are the Boundary Integral Method, the Immersed Boundary Method, and the Fictitious Domain Method (Li et al., 2017).

To make matters even more complicated, the mechanism of the blood clot formation strongly depends on the following three processes: Receptor-Ligand Binding, Platelet Activation and the Coagulation Cascade. Specifically, the initiation of thrombus development starts with tethering of circulating platelets onto the exposed subendothelial layer where a blood vessel is injured. This process involves bonding between the various receptors on the platelet surfaces to the extravascular proteins, such as the von Willebrand Factor. It is typically modeled using a Monte Carlo approach called Adhesive Dynamics, while the rate of the receptor-ligand bond formation and breakage are determined by the Bell Model that calculates the probability of dissociation events occurring over a specific timespan.

Once the platelets have been recruited to the injury site, they undergo a metamorphosis that is generally described as “activation.” During this process, the membrane receptors transmit signals to the inside of the cells, which results in the dumping of chemical agonists stored in the internal vesicles called lysosomes and granules (e.g., dense and alpha). The release of these molecules then activates other neighboring platelets, which ultimately leads them to becoming “stickier” and compacting into the blood clot’s body. Unfortunately, the bottom-up description of the process contains numerous unknowns (Dolan and Diamond, 2014). For this reason, it has been instead described via a top-down Neural Network approach, which was trained on patient-specific experimental data (Flamm et al., 2012).

**FIGURE 12:**
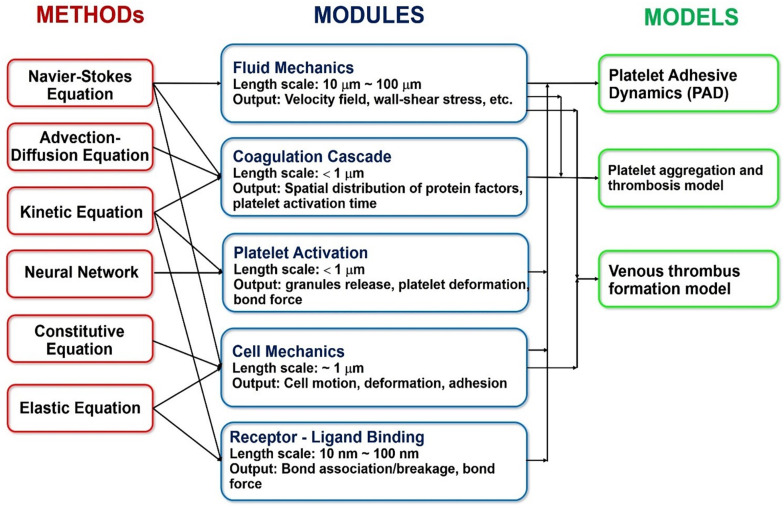
Overview of the specific methods, modules and models commonly used in the state-of-the-art multiscale platelet hemodynamics and thrombus development studies. (Adopted with permission from Wang and King, 2012).

Lastly, the coagulation cascade is a system of coupled biochemical reactions with two different initiation pathways, which both ultimately lead to the polymerization of soluble fibrinogen (a blood plasma protein) into an insoluble fibrin mesh that holds the clot together. The kinetic portion of the cascade involves 34 differential equations, with 42 rate constants, that cumulatively account for 27 independent equilibrium expressions and fates of 34 chemical species (Hockin et al., 2002). Additionally, the mass transport of these species must be tracked under the flow conditions experienced in the cardiovascular system (which involves Knudsen diffusion within the porous clot). Figure 12 summarizes the coupling of the various hemodynamics submodules, as well as the methods used to solve them.

### Module Personalization

**FIGURE 13:**
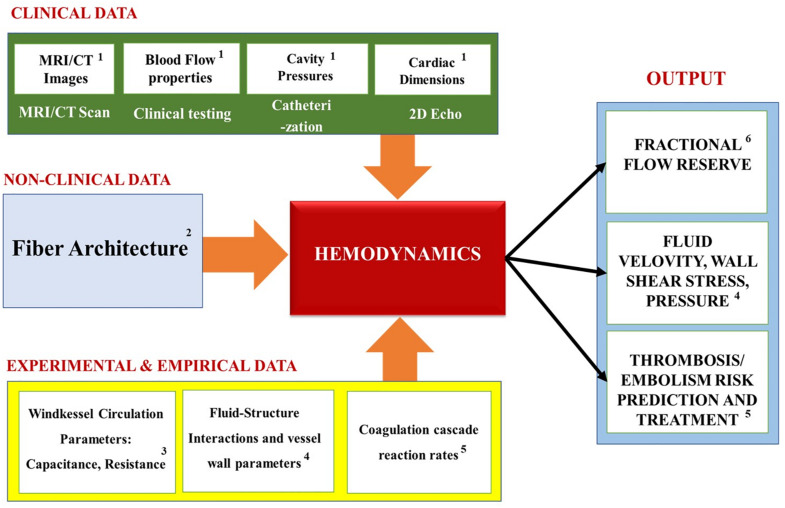
Summary of the Hemodynamics modules’ inputs, outputs and applications. Superscripts in the figure correspond to the following references: ^1^Kim et al., 2009, 2010; Kung et al., 2014; Bonfanti et al., 2019; ^2^Tang et al., 2010; Choi et al., 2015; ^3^Kung et al., 2014; Grande Gutierrez et al., 2017; ^4^Kim et al., 2010; Bonfanti et al., 2019; ^5^Seo et al., 2016; Harfi et al., 2017; ^6^Koo et al., 2011; Zhang et al., 2014; Min et al., 2015.

Table 3 summarizes the most common hemodynamics module personalization approaches encountered in the recent IBM works, while Figure 13 maps the relationships between the module’s inputs, outputs and applications. Similarly to the previous modules, the macroscopic geometry of the heart, and that of the surrounding blood vessels, is commonly obtained for use in the Hemodynamics modules via the *in vivo* imaging techniques (such as MRI and CT). Additionally, for the body’s circulation, the parameters values for the Windkessel model (i.e. ‘*R’* and ‘*C’* elements) are typically chosen to match the patient specific cardiac output, flow waveforms and pressure pulses (Kim et al., 2009, 2010; Kung et al., 2014) obtained via contrast-enhanced CT scans, Doppler ultrasound scanning and invasive blood pressure measurements (IBPM). Particularly, the Doppler ultrasonography allows the measurement of the cardiac output and heart rate; and the IBPM allows to measure the systolic and diastolic pressures (Bonfanti et al., 2019). Specifically, they are first chosen based on literature data (Segers et al., 2002; Kim et al., 2009) and are then iterated until the calculated flow parameters match the subject’s unique physiological profile (Kim et al., 2009).

**TABLE 3:** A recent literature survey of how the Hemodynamics modules are typically personalized using image-based information.

**Imaging method**	**Personalized Information from Imaging**	**Mapping of the Personalized Information from the Imaging to the Module’s Inputs**	**Citation**
CT	Coronary arteries geometry	Geometry was personalized; parameter values for coronary model were obtained from literature; parameter values of lumped heart model (for the inlet) and Windkessel models (for the outlet) were adjusted to match subject-specific cardiac output and pulse pressure.	Kim et al., 2010
MRI	Coronary arteries geometry	Geometry was personalized; parameter values of lumped heart model (for the inlet) and Windkessel models (for the outlet) were adjusted to match subject-specific flow distribution and measured brachial artery pulse pressure.	Kim et al., 2009
CT angiogram	Right coronary artery with aneurysmal region	Geometry was personalized; Windkessel model’ parameters were adjusted to match subject-specific flow distribution and pressure at outlet boundary of the coronary artery.	Kung et al., 2014
Phase contrast MRI	3-component flow velocity at two slice locations in the coronary aneurysm geometry		
Contrast-enhanced CT	Aortic dissection geometry	Geometry was personalized; inlet flowrate was obtained by adjusting a typical ascending aorta blood flow waveform to match the patient-specific hemodynamic data including cardiac output, heat-rate and systolic-to-diastolic duration ratio; for the outlet, Windkessel models parameters were adjusted to achieve patient-specific physiological flow distribution at each outlet, and to obtain the measured systolic and diastolic pressures at the inlet.	Bonfanti et al., 2019
Doppler ultrasonography	Cardiac output and heart rate		
CT angiogram	Coronary arteries geometry	Geometry was personalized; parameter values of lumped heart model (for the inlet) and Windkessel models (for the outlet) were adjusted to match subject-specific flow distribution and measured aortic pressure.	Grande Gutierrez et al., 2017
4D cardiac CT images and echocardiogram	Whole-heart geometry with kinematics of left ventricle lumen: heart rate, EDV, ESV, EF, stroke volume.	Geometry was personalized; the immersed boundary-based method is used to match the patient-specific kinematics of the left ventricular lumen including heart rate, EDV, ESV, EF and stroke volume.	Harfi et al., 2017

Unfortunately, due to the enormous complexities and computational costs of the heart modeling, oversimplifications are common in the personalization of the blood properties within the Hemodynamics modules. Given that it is typically assumed to be a Newtonian (or weakly non-Newtonian) fluid, it effectively does not contain discrete blood cells, such as the platelets or the erythrocytes. This, in turn, means that only simple flow properties, like fluid viscosity can be personalized to the subject. Consequently, the patient-specific biology of these cells (e.g., thrombotic propensity, sickle cell anemia, etc.) are omitted. Yet, in the non-cardio blood modeling, attempts at personalizing such functionality and disorders have been made: for example, the application of the Neural Networks trained on the patient-specific experimental data in order to phenotype the platelet activation (Flamm et al., 2012). However, we could not find an example of such an extensive blood personalization in the cardiovascular modeling literature.

### Module Outputs and Applications

Figure 13 summarizes the inputs, outputs and applications of the cardiac hemodynamics module. Overall, the models that use the simplified hemodynamics formulation tend to calculate the blood flow parameters, like the pressure-volume relationship of the cardiac cycle. These, in turn, help to elucidate quantities that are key to understanding the heart dysfunctions: such as the end-diastolic and the end-systolic pressure volume relationships. Additionally, Fractional Flow Reserve, which is defined as the pressure difference across a coronary artery stenosis (e.g., a narrowing due to atherosclerosis), can be calculated to determine the likelihood that the latter would impede oxygen delivery to the heart and lead to a myocardial ischemia. Furthermore, the biomechanical models measure “compliance,” which is the ability of the blood vessel walls to stretch in order to accommodate an increasing amount of blood; and “resistance” (defined as the ratio of the pressure drop and the flow change across the segment) that the blood flow experiences due to viscous stresses and constrictions by the blood vessels. Most importantly, the biomechanics-hemodynamics modeling can be used to predict the left ventricular ejection fraction (LVEF) - a main indicator of HF, which is expressed as a percentage of how much blood the left ventricle pumps out with each contraction. Lastly, such models can be used to investigate the effects of the blood pumping assist devices (e.g., LVAD) on the cardiac function, which may otherwise be too difficult or costly to study experimentally.

Conversely, the models that use the thrombogenesis hemodynamics module tend to try to assess the propensity of clot formation at (or near) the heart, based on parameters that are related to platelet activation: such as the Wall Shear Rate; the blood Residence Time Near a Damaged Tissue; the Ejection Fraction (i.e., the percentage of the blood leaving the left ventricle each time it contracts); Washout Ratio (i.e., the ratio of delayed ejection volume to the total ventricular blood at the beginning of the cycle). Additionally, some of these models simulate how drugs and clot breakup devices (e.g., Vena cava filters) help to protect the heart from pathogenic events. Overall, this is the most computationally expensive model type, due to the complexity of the thrombogenesis/embolism processes (which are themselves still being actively investigated) (Wang and King, 2012).

### Module Personalization Example

The following is a discussion of a representative example of the personalized hemodynamics modeling applied to predicting the thrombosis risk in patients with Kawasaki disease (KD) (Grande Gutierrez et al., 2019). Thrombosis is a major complication associated with coronary artery aneurysms (CAAs) resulting from the KD. In this research, the aneurysm hemodynamics were investigated for thrombotic risk stratification in ten KD patients, and were compared to the standard clinical guidelines for anticoagulation therapy. The patient-specific models were generated from MRI data by performing an angiography of: the heart, the main coronary arteries (right, left main, left anterior descending and circumflex), and the aorta and its arch branches (the brachiocephalic artery, the left common carotid artery and the left subclavian artery). This was done via the injection of gadolinium-based contrast with a cardiac and respiratory-gated 3D TrueFISP sequence. Computational hemodynamic simulations were then performed in the reconstructed anatomical model using SimVascular software (Grande Gutierrez et al., 2019). The pulsatile flow, deformable arterial walls and Windkessel parameters were tuned to match the patient-specific arterial pressure and cardiac output. Local hemodynamics variables were derived from the simulation results, including the time-averaged wall shear stress, low wall shear stress exposure and blood residence time. These variables were then used to develop a framework for predicting the thrombosis risk. Although platelet activation and aggregation are typically associated with regions of higher fluid shear (Casa et al., 2015) and longer blood stagnation (Hathcock James, 2006), this study showed that a combination of low shear stress coupled with a high residence time correlated to thrombus formation in the KD CAAs patients. Furthermore, it was shown that the prediction of the thrombotic risk using the hemodynamic variables was validated with a higher sensitivity and specificity in comparison with the standard clinical metrics. In conclusion, this type of personalized computational modeling can be used to provide a non-invasive thrombotic risk stratification that is more accurate than the current clinical approaches. This, in turn, can assist the long-term medical management of the KD patients with the CAAs.

## Summary and Conclusion

Most of the published cardiovascular modeling reviews are typically oriented at an expert audience, which makes it difficult for the outsiders to understand the full medical potential of these methods. One of the barriers to penetrating the field is that these works tend to focus on just one or two specific aspects of the simulation approach at a time: such as imaging (Weese et al., 2013; Lamata et al., 2014; Watson et al., 2018), electrophysiology (Lopez-Perez et al., 2015; Rodriguez et al., 2015; Beheshti et al., 2016; Gray and Pathmanathan, 2018; Ni et al., 2018), biomechanics (Wang et al., 2015; Chabiniok et al., 2016), hemodynamics (Zhong et al., 2018), electro-biomechanical coupling (Trayanova, 2011, 2012; Tobon-Gomez et al., 2013; Niederer et al., 2019b), biomechanics-hemodynamics coupling (Tang et al., 2010; Sun et al., 2014), etc. In contrast, our manuscript provides a “big picture” overview of the components that these models are built from; their mechanisms, inputs, outputs and connecting pipelines; the underlying physiological processes that they represent; their image-based personalization to the individual patient’s unique anatomy; and their applications to the different cardiovascular disease understanding and treatments.

**FIGURE 14:**
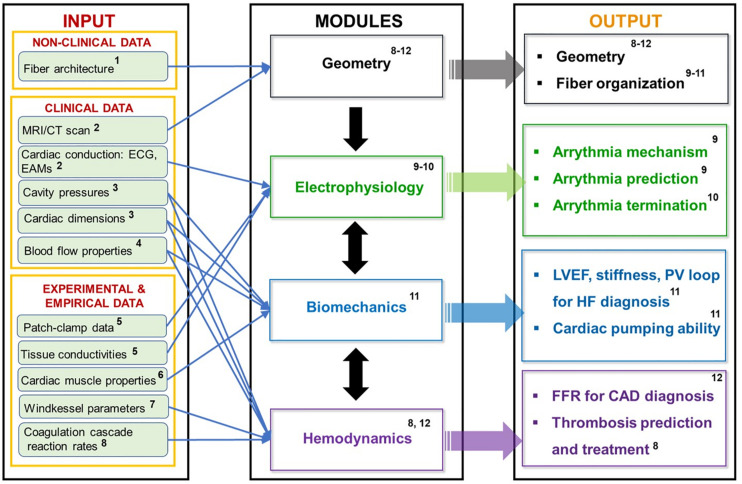
Summary of cardiovascular IBM’s modules and their respective inputs and outputs with corresponding references: ^1^Krishnamurthy et al., 2013; Lopez-Perez et al., 2015; ^2^Krishnamurthy et al., 2013; Cardenes et al., 2014; Kayvanpour et al., 2015; Lopez-Perez et al., 2015; ^3^Krishnamurthy et al., 2013; Grande Gutierrez et al., 2017; Finsberg et al., 2018; ^4^Meoli et al., 2015; Bonfanti et al., 2019; ^5^Arevalo et al., 2016; Trayanova et al., 2017; Lopez-Perez et al., 2019; ^6^Krishnamurthy et al., 2013; Finsberg et al., 2018; Palit et al., 2018; ^7^Kung et al., 2014; Grande Gutierrez et al., 2017; Bonfanti et al., 2019; ^8^Choi et al., 2015; Seo et al., 2016; Harfi et al., 2017; ^9^Arevalo et al., 2016; Deng et al., 2016; Trayanova and Chang, 2016; Prakosa et al., 2018; ^10^Arevalo et al., 2016; Trayanova et al., 2017; Deng et al., 2019; ^11^Voorhees and Han, 2015; Walmsley et al., 2017; Lee et al., 2018; ^12^Koo et al., 2011; Min et al., 2012, 2015; Zhang et al., 2014; Min et al., 2015.

As a part of our review, it was found that although this type of modeling holds a tremendous potential for revolutionizing personalized cardiovascular medicine, it is still in its infancy (with HeartFlow being the only commercially available product). Furthermore, due to the slow speed of high-resolution imaging, most of the IBM in academia still rely on scans of dead hearts (as opposed to beating ones). This, however, is expected to change, as the imaging speeds of mCT and MRI are increased. As far as the physics being modeled, the modules, their inputs and outputs are summarized in Figure 14. The simplest application of the cardiovascular IBM is to the study of arrythmias, which simulates the propagation of electrical impulses through the myocardium, while ignoring the biomechanics and hemodynamics. At the subcellular level, these models calculate transfer of ions across the cell membrane channels, while at the macro level the transfer of potential between the cells is modeled as a diffusive process. Conduction irregularities, ablation targets, and effects of defibrillation are just some of the outputs provided by the electrophysiological models.

The more complex models account for the biomechanical processes occurring in the myocardium. These models are focused on how abnormalities in the tissue morphology and stiffness affect the pumping efficiency of the heart. At the subcellular level, they simulate how the electrically driven calcium signaling governs the crossbridge mechanism of the active tension generation via the actin-myosin interactions in the cells’ sarcomeres. Additionally, the solid mechanics calculation also involves the passive stress of the myocardium, which represents the stress-strain relationship of the cardiac fibers without the electrical stimulation. The resulting tissue deformation is then backwards-coupled to the electrophysiology, since the stretching of the cells can change the gap junction distance to their neighbors (which results in changes to the ionic conductivity).

Lastly, these models are coupled to the hemodynamics calculations via fluid-structure interactions. If only the blood flow without the clot formation is of interest, then the structure of the fluid is approximated to be homogeneous and Newtonian; while the rest of the body’s circulation is introduced as an oscillating pressure boundary condition that is assumed to behave like a simple RC circuit. This type of model can provide insight into HF due to deformities and obstructions, as well as allow for virtual design and testing of assisted pumping devices. On the other hand, if clotting information is necessary, the blood must be treated as a suspension of deformable particles, with receptor ligand interactions, intra-cellular signaling, and coupled biochemical reactions representing the coagulation cascade. Although a lot more involved, this type of model can be useful for antithrombotic drug development and design of clot breakup devices (e.g., Vena cava filters) meant to protect the heart. However, for simplicity most cardiovascular IBM do not account for the hydrodynamics and biomechanics of the individual blood cells. Likewise, the coagulation cascade and the platelet activation, both of which are central to thrombogenesis, are oversimplified relative to the state-of-the-art within the non-cardiovascular blood modeling field.

Overall, the cardiovascular IBM is expected to become more mainstream as the computational and imaging technologies advance. This could potentially revolutionize how cardiovascular medicine is done in the future. Yet, significant improvements are still required to personalize the models more. For example, a common limitation across all of the modules is that the myocardium (e.g., conductivity and tissue stiffness) and the blood (e.g., hematocrit, coagulation cascade and platelet activation kinetics and deficiencies) properties that they use are typically estimated based on empirical measurements performed *ex vivo* and using samples that are not derived from the same individual (or even from the same species for that matter). Therefore, better imaging methods need to be developed, such that these properties could be estimated by scanning the individual patient. Furthermore, a finer image resolution is needed to capture the individualized variations in the conductive and contractile fibers, and their junctions. Likewise, the computational methods need to improve their modeling of the intra-cellular processes (e.g., the crossbridge mechanism) for the cardiovascular IBM to become more physiologically representative and adopted by the mainstream clinical market. However, given the fast pace of the technological progress, the near future impact of the IBM on the cardiovascular medicine is imminent.

## Author Contributions

TN and OK devised the manuscript, performed review of literature, and wrote the manuscript. RV provided the feedback and edits on all aspects of the manuscript. All the authors contributed to the article and approved the submitted version.

## Conflict of Interest

The authors declare that the research was conducted in the absence of any commercial or financial relationships that could be construed as a potential conflict of interest.

## Glossary

### Action Potential

A brief reversal (i.e., “depolarization” increase from a negative resting state followed by “repolarization” down to the original levels) in the voltage polarity of cardiomyocytes, which is generated by ion (e.g., Ca++, Na+, and K+) exchange across specialized channels in the cell membrane. The propagation of this electrical impulse through the heart tissue occurs via currents of the Ca++ and K+ ions moving through the gap junctions from the activated cells to their resting neighbors. This leads to the eventual contraction of the cardiac fibers along the conduction pathway.

### Activation Rate Gradient

An arrythmia measure indicating a difference in the speed with which cardiomyocytes in the epicardial and in the endocardial tissues activate (i.e., depolarize/repolarize) in response to applied external stimuli (e.g., electrodes, needles, heat, blocking ion channels via medications, etc.) during Ventricular Fibrillation studies.

### Bidomain Formulation

A mathematical framework that is used in Electrophysiology modeling to simulate the propagation of the *Action Potential* (see definition) through the myocardium. As opposed to the *Monodomain Formulation* (see definition), it considers both the intra- and the extra- cellular domains of the ion exchange across the cardiomyocyte membrane separately.

### Cauchy–Green (Left and Right) Tensors

Rotation-independent measures of the material (e.g., myocardium tissue) deformation, in a spatial reference and in the object’s coordinate systems, respectively. They are often used when describing the passive properties of hyperelastic materials, such as the diastolic cardiac dysfunction.

### Conductivity Tensor

A mathematical quantity that describes the electrical conductivity of the myocardium. This property is highly dependent on the orientation of the fibers that the conductive heart cells are arranged into within the tissue. For this reason, the effective conductivity of the myocardium differs, depending on the direction of the current flow. Therefore, the tensor encompasses 9 total conductivity values: three of them represent directions along each of the principle axes, while the other six express the correlation of the conductivity between each pair of the principal directions.

### Deformation Gradient Tensor

A mathematical quantity that describes a shape change (e.g., stretch), as well as overall rotation, relative to an initial state of a material (e.g., cardiac fiber structure). It holds information about the difference in the current locations of neighboring cardiomyocytes and is unity when they are displaced equally (i.e., for no deformation).

### Diffusion Tensor

A mathematical quantity calculated in magnetic resonance imaging to visualize structural arrangements (e.g., fibers, sheets) of the cardiomyocytes within the myocardium. It is based on at least 6 unique (plus one baseline) measurements of how the diffusion of water molecules is restricted or biased in different directions by the structural obstructions that they encounter during their motion in the tissues of the hearts. Therefore, the tensor encompasses three main elements that represent diffusion coefficients along each of the principal axes, while the off-diagonal terms reflect the correlation of random motions between each pair of principal directions.

### Determinant of the Deformation Gradient Tensor

A scalar value corresponding to the ratio of the deformed to the undeformed volume, which is computed from the elements of the *Deformation Gradient Tensor* (see definition) in order to quantify the amount of transformation occurring in the cardiac geometry.

### Invariant

A property of a mathematical quantity which remains unchanged after an application of an operation or a transformation. For example, when calculating strain and stress of the cardiac fibers it is important that the resulting principal values remain the same, regardless of the coordinate system chosen for their calculation or measurement.

### Membrane Capacitance

A constant that describes the relationship between the voltage across the membrane of a cell and the ionic charge that builds up on its interior and exterior surfaces. In cardiomyocytes it effectively determines how quickly the *Action Potential* (see definition) can travel through the myocardium. It is therefore useful for both modeling and understanding their excitability and pathological conditions.

### Monodomain Formulation

A mathematical framework that is used in Electrophysiology modeling to simulate the propagation of the *Action Potential* through the myocardium. As opposed to the *Bidomain Formulation* (see definition), it does not consider both the intra- and the extra- cellular domains of the ion exchange across the cardiomyocyte membrane. Instead, the framework is expressed in terms of a transmembrane potential, which assumes that the intracellular and extracellular ion diffusivities are proportional to each other.

### Neural Network

A set of artificial intelligence algorithms, modeled loosely after how the human brain learns, that are designed to recognize patterns within complex datasets. In cardiovascular modeling specifically, they are used to represent processes that are too difficult to describe mathematically using first-principle methods: for example, platelet “activation” – a cascade of cell membrane surface receptor activation, intracellular signaling, dumping of granules, etc. – in the context of thrombosis.

### Stored (a.k.a., Strain) Energy Density Function

Energy per unit volume of a material temporarily deformed by an applied force, like a coiled spring or a stretched elastic band. This quantity is commonly used to formulate constitutive force-deformation relationships that characterize the spatial and temporal variations in the orthotropic (i.e., different in the axial, radial, and circumferential directions) properties of the myocardium. It is largely dominated by the structural arrangements of the myocardial fibers in the heart tissue.

## References

[B1] Antzelevitch C. (2001). Basic mechanisms of reentrant arrhythmias. *Curr. Opin. Cardiol.* 16 1–7. 10.1097/00001573-200101000-00001 11124712

[B2] Appleton B. Qing W. Crozier S. Feng L. Wilson S. Ling X. (2005). “An electrical heart model incorporating real geometry and motion,” in *Proceedings of the 27th Annual Conference IEEE Engineering in Medicine and Biology*, Shanghai, 345–348. 10.1109/IEMBS.2005.1616415 17282184

[B3] Arevalo H. J. Vadakkumpadan F. Guallar E. Jebb A. Malamas P. Wu K. C. (2016). Arrhythmia risk stratification of patients after myocardial infarction using personalized heart models. *Nat. Commun.* 7:11437. 10.1038/ncomms11437 27164184 PMC4866040

[B4] Ashikaga H. Arevalo H. Vadakkumpadan F. Blake R. C. Bayer J. D. Nazarian S. (2013). Feasibility of image-based simulation to estimate ablation target in human ventricular arrhythmia. *Heart Rhythm* 10 1109–1116. 10.1016/j.hrthm.2013.04.015 23608593 PMC3735782

[B5] Aslanidi O. V. Nikolaidou T. Zhao J. Smaill B. H. Gilbert S. H. Holden A. V. (2013). Application of micro-computed tomography with iodine staining to cardiac imaging, segmentation, and computational model development. *IEEE Trans. Med. Imaging* 32 8–17. 10.1109/TMI.2012.2209183 22829390 PMC3493467

[B6] Avazmohammadi R. Soares J. S. Li D. S. Raut S. S. Gorman R. C. Sacks M. S. (2019). A contemporary look at biomechanical models of myocardium. *Annu. Rev. Biomed. Eng.* 21 417–442. 10.1146/annurev-bioeng-062117-121129 31167105 PMC6626320

[B7] Bayer J. D. Blake R. C. Plank G. Trayanova N. A. (2012). A novel rule-based algorithm for assigning myocardial fiber orientation to computational heart models. *Ann. Biomed. Eng.* 40 2243–2254. 10.1007/s10439-012-0593-5 22648575 PMC3518842

[B8] Beheshti M. Umapathy K. Krishnan S. (2016). Electrophysiological cardiac modeling: a review. *Crit Rev Biomed Eng.* 44 99–122. 10.1615/CritRevBiomedEng.2016016454 27652454

[B9] Behradfar E. Nygren A. Vigmond E. J. (2014). The role of Purkinje-myocardial coupling during ventricular arrhythmia: a modeling study. *PLoS One* 9:e88000. 10.1371/journal.pone.0088000 24516576 PMC3917859

[B10] Bestel J. Clément F. Sorine M. (2001). “A biomechanical model of muscle contraction,” in *Medical Image Computing and Computer-Assisted Intervention – MICCAI 2001*, eds Niessen W. J. Viergever M. A. (Berlin: Springer), 1159–1161. 10.1007/3-540-45468-3_143

[B11] Bishop M. J. Plank G. (2011). Bidomain ECG simulations using an augmented monodomain model for the cardiac source. *Proc. IEEE Trans. Biomed. Eng.* 58 2297–2307. 10.1109/TBME.2011.2148718 21536529 PMC3378475

[B12] Bishop M. J. Plank G. Burton R. A. Schneider J. E. Gavaghan D. J. Grau V. (2010). Development of an anatomically detailed MRI-derived rabbit ventricular model and assessment of its impact on simulations of electrophysiological function. *Am. J. Physiol. Heart Circ. Physiol.* 298 H699–H718. 10.1152/ajpheart.00606.2009 19933417 PMC2822578

[B13] Bonfanti M. Franzetti G. Maritati G. Homer-Vanniasinkam S. Balabani S. Díaz-Zuccarini V. (2019). Patient-specific haemodynamic simulations of complex aortic dissections informed by commonly available clinical datasets. *Med. Eng. Phys.* 71 45–55. 10.1016/j.medengphy.2019.06.012 31257054

[B14] Boyle P. M. Masse S. Nanthakumar K. Vigmond E. J. (2013). Transmural IK(ATP) heterogeneity as a determinant of activation rate gradient during early ventricular fibrillation: mechanistic insights from rabbit ventricular models. *Heart Rhythm* 10 1710–1717. 10.1016/j.hrthm.2013.08.010 23948344 PMC3826179

[B15] Boyle P. M. Zahid S. Trayanova N. A. (2016). Towards personalized computational modelling of the fibrotic substrate for atrial arrhythmia. *Europace* 18 (Suppl. 4), iv136–iv145. 10.1093/europace/euw358 28011841 PMC5841887

[B16] Bruegmann T. Boyle P. M. Vogt C. C. Karathanos T. V. Arevalo H. J. Fleischmann B. K. (2016). Optogenetic defibrillation terminates ventricular arrhythmia in mouse hearts and human simulations. *J. Clin. Invest.* 126 3894–3904. 10.1172/JCI88950 27617859 PMC5096832

[B17] Burton R. A. Plank G. Schneider J. E. Grau V. Ahammer H. Keeling S. L. (2006). Three-dimensional models of individual cardiac histoanatomy: tools and challenges. *Ann. N. Y. Acad. Sci.* 1080 301–319. 10.1196/annals.1380.023 17132791 PMC3313659

[B18] Cabo C. Boyden P. A. (2003). Electrical remodeling of the epicardial border zone in the canine infarcted heart: a computational analysis. *Am. J. Physiol. Heart Circ. Physiol.* 284 H372–H384. 10.1152/ajpheart.00512.2002 12388240

[B19] Cardenes R. Sebastian R. Berruezo A. Camara O. (2014). “Inverse estimation of ventricular Purkinje tree pathways from sequences of depolarization,” in *Proceedings of the 2014 Conference on Computing in Cardiology*, Cambridge, MA, 677–680.

[B20] Casa L. D. C. Deaton D. H. Ku D. N. (2015). Role of high shear rate in thrombosis. *J. Vasc. Surg.* 61 1068–1080. 10.1016/j.jvs.2014.12.050 25704412

[B21] CDC (2019). *Heart Disease Facts [Online].* Available online at: https://www.cdc.gov/heartdisease/facts.htm (accessed on 26 December 2019)

[B22] Chabiniok R. Wang V. Y. Hadjicharalambous M. Asner L. Lee J. Sermesant M. (2016). Multiphysics and multiscale modelling, data–model fusion and integration of organ physiology in the clinic: ventricular cardiac mechanics. *Interf. Focus* 6:20150083. 10.1098/rsfs.2015.0083 27051509 PMC4759748

[B23] Cheng Y. Oertel H. Schenkel T. (2005). Fluid-structure coupled CFD simulation of the left ventricular flow during filling phase. *Ann. Biomed. Eng.* 33 567–576. 10.1007/s10439-005-4388-9 15981858

[B24] Chnafa C. Mendez S. Nicoud F. (2014). Image-based large-eddy simulation in a realistic left heart. *Comput. Fluids* 94 173–187. 10.1016/j.compfluid.2014.01.030

[B25] Choi Y. J. Constantino J. Vedula V. Trayanova N. Mittal R. (2015). A new MRI-based model of heart function with coupled hemodynamics and application to normal and diseased canine left ventricles. *Front. Bioeng. Biotechnol.* 3:140. 10.3389/fbioe.2015.00140 26442254 PMC4585083

[B26] Craft (2019). *HeartFlow [Online].* Available online at: https://craft.co/heartflow (accessed on 26 December 2019).

[B27] Dass S. Suttie J. J. Piechnik S. K. Ferreira V. M. Holloway C. J. Banerjee R. (2012). Myocardial tissue characterization using magnetic resonance noncontrast t1 mapping in hypertrophic and dilated cardiomyopathy. *Circ. Cardiovasc. Imaging* 5 726–733. 10.1161/CIRCIMAGING.112.976738 23071146

[B28] Decker K. F. Rudy Y. (2010). Ionic mechanisms of electrophysiological heterogeneity and conduction block in the infarct border zone. *Am. J. Physiol. Heart Circ. Physiol.* 299 H1588–H1597. 10.1152/ajpheart.00362.2010 20709867 PMC2993197

[B29] Deng D. Arevalo H. J. Prakosa A. Callans D. J. Trayanova N. A. (2016). A feasibility study of arrhythmia risk prediction in patients with myocardial infarction and preserved ejection fraction. *Europace* 18 (Suppl. 4), iv60–iv66. 10.1093/europace/euw351 28011832 PMC5225965

[B30] Deng D. Jiao P. Ye X. Xia L. (2012). An image-based model of the whole human heart with detailed anatomical structure and fiber orientation. *Comput. Math. Methods Med.* 2012:891070. 10.1155/2012/891070 22952559 PMC3431151

[B31] Deng D. Prakosa A. Shade J. Nikolov P. Trayanova N. A. (2019). Sensitivity of ablation targets prediction to electrophysiological parameter variability in image-based computational models of ventricular tachycardia in post-infarction patients. *Front. Physiol.* 10:628. 10.3389/fphys.2019.00628 31178758 PMC6543853

[B32] Dokos S. Smaill B. H. Young A. A. LeGrice I. J. (2002). Shear properties of passive ventricular myocardium. *Am. J. Physiol. Heart Circ. Physiol.* 283 H2650–H2659. 10.1152/ajpheart.00111.2002 12427603

[B33] Dolan A. T. Diamond S. L. (2014). Systems modeling of Ca(2+) homeostasis and mobilization in platelets mediated by IP3 and store-operated Ca(2+) entry. *Biophys. J.* 106 2049–2060. 10.1016/j.bpj.2014.03.028 24806937 PMC4017292

[B34] Donati F. Myerson S. Bissell M. M. Smith N. P. Neubauer S. Monaghan M. J. (2017). Beyond Bernoulli: improving the accuracy and precision of noninvasive estimation of peak pressure drops. *Circ. Cardiovasc. Imaging* 10:e005207. 10.1161/CIRCIMAGING.116.005207 28093412 PMC5265685

[B35] Durrer D. Van Dam R. T. H. Freud G. E. Janse M. J. Meijler F. L. Arzbaecher R. C. (1970). Total excitation of the isolated human heart. *Circulation* 41 899–912. 10.1161/01.CIR.41.6.899 5482907

[B36] FDA (2019). *510(k) Premarket Notification.* Silver Spring, MD: FDA.

[B37] Finsberg H. Xi C. Tan J. L. Zhong L. Genet M. Sundnes J. (2018). Efficient estimation of personalized biventricular mechanical function employing gradient-based optimization. *Int. J. Numer. Method Biomed. Eng.* 34:e2982. 10.1002/cnm.2982 29521015 PMC6043386

[B38] Flachskampf F. A. Weyman A. E. Guerrero J. L. Thomas J. D. (1990). Influence of orifice geometry and flow rate on effective valve area: an in vitro study. *J. Am. Coll. Cardiol.* 15 1173–1180. 10.1016/0735-1097(90)90260-V 2312974

[B39] Flamm M. H. Colace T. V. Chatterjee M. S. Jing H. Zhou S. Jaeger D. (2012). Multiscale prediction of patient-specific platelet function under flow. *Blood* 120 190–198. 10.1182/blood-2011-10-388140 22517902 PMC3390957

[B40] Frangi A. F. Rueckert D. Schnabel J. A. Niessen W. J. (2002). Automatic construction of multiple-object three-dimensional statistical shape models: application to cardiac modeling. *IEEE Trans. Med. Imaging* 21 1151–1166. 10.1109/TMI.2002.804426 12564883

[B41] Fritz T. Wieners C. Seemann G. Steen H. Dossel O. (2014). Simulation of the contraction of the ventricles in a human heart model including atria and pericardium. *Biomech. Model. Mechanobiol.* 13 627–641. 10.1007/s10237-013-0523-y 23990017

[B42] Goldberger J. J. Buxton A. E. Cain M. Costantini O. Exner D. V. Knight B. P. (2011). Risk stratification for arrhythmic sudden cardiac death: identifying the roadblocks. *Circulation* 123 2423–2430. 10.1161/CIRCULATIONAHA.110.959734 21632516

[B43] Grande Gutierrez N. Kahn A. Burns J. C. Marsden A. L. (2017). Computational blood flow simulations in Kawasaki disease patients: insight into coronary artery aneurysm hemodynamics. *Glob. Cardiol. Sci. Pract.* 2017:e201729. 10.21542/gcsp.2017.29 29564350 PMC5856960

[B44] Grande Gutierrez N. Mathew M. McCrindle B. W. Tran J. S. Kahn A. M. Burns J. C. (2019). Hemodynamic variables in aneurysms are associated with thrombotic risk in children with Kawasaki disease. *Int. J. Cardiol.* 281 15–21. 10.1016/j.ijcard.2019.01.092 30728104 PMC6511338

[B45] Gray R. A. Pathmanathan P. (2018). Patient-specific cardiovascular computational modeling: diversity of personalization and challenges. *J. Cardiovasc. Transl. Res.* 11 80–88. 10.1007/s12265-018-9792-2 29512059 PMC5908828

[B46] Guccione J. M. Salahieh A. Moonly S. M. Kortsmit J. Wallace A. W. Ratcliffe M. B. (2003). Myosplint decreases wall stress without depressing function in the failing heart: a finite element model study. *Ann. Thorac. Surg.* 76 1171–1180. 10.1016/s0003-4975(03)00731-8 14530007

[B47] Harfi T. T. Seo J. H. Yasir H. S. Welsh N. Mayer S. A. Abraham T. P. (2017). The E-wave propagation index (EPI): a novel echocardiographic parameter for prediction of left ventricular thrombus. Derivation from computational fluid dynamic modeling and validation on human subjects. *Int. J. Cardiol.* 227 662–667. 10.1016/j.ijcard.2016.10.079 27838120

[B48] Hathcock James J. (2006). Flow effects on coagulation and thrombosis. *Arterioscl. Thromb. Vasc. Biol.* 26 1729–1737. 10.1161/01.ATV.0000229658.76797.30 16741150

[B49] HeartFlow (2019). *Transforming the Diagnosis and Management of Coronary Artery Disease Worldwide.* Available online at: https://www.heartflow.com/ (accessed on 13 December 2019)

[B50] Hockin M. F. Jones K. C. Everse S. J. Mann K. G. (2002). A model for the stoichiometric regulation of blood coagulation. *J. Biol. Chem.* 277 18322–18333. 10.1074/jbc.M201173200 11893748

[B51] Hodgkin A. L. Huxley A. F. (1952). A quantitative description of membrane current and its application to conduction and excitation in nerve. *J. Physiol.* 117 500–544. 10.1113/jphysiol.1952.sp004764 12991237 PMC1392413

[B52] Holmes A. A. Scollan D. F. Winslow R. L. (2000). Direct histological validation of diffusion tensor MRI in formaldehyde-fixed myocardium. *Magn. Reson. Med.* 44 157–161. 10.1002/1522-2594(200007)44:1<157::aid-mrm22<3.0.co;2-f 10893534

[B53] Holzapfel G. A. Ogden R. W. (2009). Constitutive modelling of passive myocardium: a structurally based framework for material characterization. *Philos. Trans. A Math. Phys. Eng. Sci.* 367 3445–3475. 10.1098/rsta.2009.0091 19657007

[B54] Hooks D. A. Tomlinson K. A. Marsden S. G. LeGrice I. J. Smaill B. H. Pullan A. J. (2002). Cardiac microstructure: implications for electrical propagation and defibrillation in the heart. *Circ. Res.* 91 331–338. 10.1161/01.res.0000031957.70034.89 12193466

[B55] Hooks D. A. Trew M. L. Smaill B. H. Pullan A. J. (2006). Do intramural virtual electrodes facilitate successful defibrillation? Model-based analysis of experimental evidence. *J. Cardiovasc. Electrophysiol.* 17 305–311. 10.1111/j.1540-8167.2006.00360.x 16643406

[B56] Huxley A. F. (1957). 6 - Muscle structure and theories of contraction. *Prog. Biophys. Biophys. Chem.* 7 255–318. 10.1016/S0096-4174(18)30128-8 13485191

[B57] Ideker R. E. Kong W. Pogwizd S. (2009). Purkinje fibers and arrhythmias. *Pac. Clin. Electrophysiol. PACE* 32 283–285. 10.1111/j.1540-8159.2008.02232.x 19272054 PMC2743071

[B58] Kayvanpour E. Mansi T. Sedaghat-Hamedani F. Amr A. Neumann D. Georgescu B. (2015). Towards personalized cardiology: multi-scale modeling of the failing heart. *PLoS One* 10:e0134869. 10.1371/journal.pone.0134869 26230546 PMC4521877

[B59] Keldermann R. H. ten Tusscher K. H. W. J. Nash M. P. Bradley C. P. Hren R. Taggart P. (2009). A computational study of mother rotor VF in the human ventricles. *Am. J. Physiol. Heart Circ. Physiol.* 296 H370–H379. 10.1152/ajpheart.00952.2008 19060124 PMC2643893

[B60] Kim H. J. Vignon-Clementel I. E. Coogan J. S. Figueroa C. A. Jansen K. E. Taylor C. A. (2010). Patient-specific modeling of blood flow and pressure in human coronary arteries. *Ann. Biomed. Eng.* 38 3195–3209. 10.1007/s10439-010-0083-6 20559732

[B61] Kim H. J. Vignon-Clementel I. E. Figueroa C. A. LaDisa J. F. Jansen K. E. Feinstein J. A. (2009). On coupling a lumped parameter heart model and a three-dimensional finite element aorta model. *Ann. Biomed. Eng.* 37 2153–2169. 10.1007/s10439-009-9760-8 19609676

[B62] Kim J. (2019). *Fast Company Names HeartFlow One of the World’s Most Innovative Companies [Online].* Available online at: https://www.businesswire.com/news/home/20190220005218/en/Fast-Company-Names-HeartFlow-World’s-Innovative-Companies/ (accessed on 13 December 2019)

[B63] Kohl P. Noble D. Hunter P. J. Kovács S. J. McQueen D. M. Peskin C. S. (2001). Modelling cardiac fluid dynamics and diastolic function. *Philos. Trans. R. Soc. Lond. Ser. A Math. Phys. Eng. Sci.* 359 1299–1314. 10.1098/rsta.2001.0832

[B64] Koo B. K. Erglis A. Doh J. H. Daniels D. V. Jegere S. Kim H. S. (2011). Diagnosis of ischemia-causing coronary stenoses by noninvasive fractional flow reserve computed from coronary computed tomographic angiograms. Results from the prospective multicenter DISCOVER-FLOW (Diagnosis of Ischemia-Causing Stenoses Obtained Via Noninvasive Fractional Flow Reserve) study. *J. Am. Coll. Cardiol.* 58 1989–1997. 10.1016/j.jacc.2011.06.066 22032711

[B65] Krishnamurthy A. Villongco C. T. Chuang J. Frank L. R. Nigam V. Belezzuoli E. (2013). Patient-specific models of cardiac biomechanics. *J. Comput. Phys.* 244 4–21. 10.1016/j.jcp.2012.09.015 23729839 PMC3667962

[B66] Krueger M. W. Schulze W. H. Rhode K. S. Razavi R. Seemann G. Dossel O. (2013a). Towards personalized clinical in-silico modeling of atrial anatomy and electrophysiology. *Med. Biol. Eng. Comput.* 51 1251–1260. 10.1007/s11517-012-0970-0 23070728

[B67] Krueger M. W. Seemann G. Rhode K. Keller D. U. J. Schilling C. Arujuna A. (2013b). Personalization of atrial anatomy and electrophysiology as a basis for clinical modeling of radio-frequency ablation of atrial fibrillation. *IEEE Trans. Med. Imaging* 32 73–84. 10.1109/TMI.2012.2201948 22665507

[B68] Kung E. Kahn A. M. Burns J. C. Marsden A. (2014). In vitro validation of patient-specific hemodynamic simulations in coronary aneurysms caused by Kawasaki disease. *Cardiovasc. Eng. Technol.* 5 189–201. 10.1007/s13239-014-0184-8 25050140 PMC4103185

[B69] Lamata P. Casero R. Carapella V. Niederer S. A. Bishop M. J. Schneider J. E. (2014). Images as drivers of progress in cardiac computational modelling. *Prog. Biophys. Mol. Biol.* 115 198–212. 10.1016/j.pbiomolbio.2014.08.005 25117497 PMC4210662

[B70] Lee A. W. C. Costa C. M. Strocchi M. Rinaldi C. A. Niederer S. A. (2018). Computational modeling for cardiac resynchronization therapy. *J. Cardiovasc. Transl. Res.* 11 92–108. 10.1007/s12265-017-9779-4 29327314 PMC5908824

[B71] Lee L. C. Wall S. T. Klepach D. Ge L. Zhang Z. Lee R. J. (2013). Algisyl-LVR with coronary artery bypass grafting reduces left ventricular wall stress and improves function in the failing human heart. *Int. J. Cardiol.* 168 2022–2028. 10.1016/j.ijcard.2013.01.003 23394895 PMC3748222

[B72] Li X. Li H. Chang H. Y. Lykotrafitis G. Em Karniadakis G. (2017). Computational biomechanics of human red blood cells in hematological disorders. *J. Biomech. Eng.* 139 0210081–02100813. 10.1115/1.4035120 PMC5395917 27814430

[B73] Lopez-Perez A. Sebastian R. Ferrero J. M. (2015). Three-dimensional cardiac computational modelling: methods, features and applications. *BioMed. Eng. Online* 14:35. 10.1186/s12938-015-0033-5 25928297 PMC4424572

[B74] Lopez-Perez A. Sebastian R. Izquierdo M. Ruiz R. Bishop M. Ferrero J. M. (2019). Personalized cardiac computational models: from clinical data to simulation of infarct-related ventricular tachycardia. *Front. Physiol.* 10:580. 10.3389/fphys.2019.00580 31156460 PMC6531915

[B75] Meoli A. Cutri E. Krishnamurthy A. Dubini G. Migliavacca F. Hsia T. Y. (2015). A multiscale model for the study of cardiac biomechanics in single-ventricle surgeries: a clinical case. *Interface Focus* 5:20140079. 10.1098/rsfs.2014.0079 25844151 PMC4342947

[B76] Mewton N. Liu C. Y. Croisille P. Bluemke D. Lima J. A. (2011). Assessment of myocardial fibrosis with cardiovascular magnetic resonance. *J. Am. Coll. Cardiol.* 57 891–903. 10.1016/j.jacc.2010.11.013 21329834 PMC3081658

[B77] Mihalef V. Ionasec R. I. Sharma P. Georgescu B. Voigt I. Suehling M. (2011). Patient-specific modelling of whole heart anatomy, dynamics and haemodynamics from four-dimensional cardiac CT images. *Interface Focus* 1 286–296. 10.1098/rsfs.2010.0036 22670200 PMC3262442

[B78] Min J. K. Koo B. K. Erglis A. Doh J. H. Daniels D. V. Jegere S. (2012). Usefulness of noninvasive fractional flow reserve computed from coronary computed tomographic angiograms for intermediate stenoses confirmed by quantitative coronary angiography. *Am. J. Cardiol.* 110 971–976. 10.1016/j.amjcard.2012.05.033 22749390

[B79] Min J. K. Taylor C. A. Achenbach S. Koo B. K. Leipsic J. Norgaard B. L. (2015). Noninvasive fractional flow reserve derived from coronary CT angiography: clinical data and scientific principles. *JACC Cardiovasc. Imaging* 8 1209–1222. 10.1016/j.jcmg.2015.08.006 26481846

[B80] Mincholé A. Zacur E. Ariga R. Grau V. Rodriguez B. (2019). MRI-based computational torso/biventricular multiscale models to investigate the impact of anatomical variability on the ECG QRS complex. *Front. Physiol.* 10:1103. 10.3389/fphys.2019.01103 31507458 PMC6718559

[B81] Mittal R. Seo J. H. Vedula V. Choi Y. J. Liu H. Huang H. H. (2016). Computational modeling of cardiac hemodynamics: current status and future outlook. *J. Comput. Phys.* 305 1065–1082. 10.1016/j.jcp.2015.11.022

[B82] Moireau P. Xiao N. Astorino M. Figueroa C. A. Chapelle D. Taylor C. A. (2012). External tissue support and fluid-structure simulation in blood flows. *Biomech. Model. Mechanobiol.* 11 1–18. 10.1007/s10237-011-0289-z 21308393

[B83] Morris P. D. Narracott A. von Tengg-Kobligk H. Silva Soto D. A. Hsiao S. Lungu A. (2016). Computational fluid dynamics modelling in cardiovascular medicine. *Heart* 102 18–28. 10.1136/heartjnl-2015-308044 26512019 PMC4717410

[B84] Negroni J. A. Lascano E. C. (2008). Simulation of steady state and transient cardiac muscle response experiments with a Huxley-based contraction model. *J. Mol. Cell Cardiol.* 45 300–312. 10.1016/j.yjmcc.2008.04.012 18550079

[B85] Ni H. Morotti S. Grandi E. (2018). A heart for diversity: simulating variability in cardiac arrhythmia research. *Front. Physiol.* 9:958. 10.3389/fphys.2018.00958 30079031 PMC6062641

[B86] Niederer S. A. Campbell K. S. Campbell S. G. (2019a). A short history of the development of mathematical models of cardiac mechanics. *J. Mol. Cell. Cardiol.* 127 11–19. 10.1016/j.yjmcc.2018.11.015 30503754 PMC6525149

[B87] Niederer S. A. Lumens J. Trayanova N. A. (2019b). Computational models in cardiology. *Nat. Rev. Cardiol.* 16 100–111. 10.1038/s41569-018-0104-y 30361497 PMC6556062

[B88] O’Hara T. Virag L. Varro A. Rudy Y. (2011). Simulation of the undiseased human cardiac ventricular action potential: model formulation and experimental validation. *PLoS Comput. Biol.* 7:e1002061. 10.1371/journal.pcbi.1002061 21637795 PMC3102752

[B89] Palamara S. Vergara C. Catanzariti D. Faggiano E. Pangrazzi C. Centonze M. (2014). Computational generation of the Purkinje network driven by clinical measurements: the case of pathological propagations. *Int. J. Numer. Methods Biomed. Eng.* 30 1558–1577. 10.1002/cnm.2689 25319252

[B90] Palit A. Bhudia S. K. Arvanitis T. N. Turley G. A. Williams M. A. (2018). In vivo estimation of passive biomechanical properties of human myocardium. *Med. Biol. Eng. Comput.* 56 1615–1631. 10.1007/s11517-017-1768-x 29479659 PMC6096751

[B91] Papadacci C. Finel V. Provost J. Villemain O. Bruneval P. Gennisson J.-L. (2017). Imaging the dynamics of cardiac fiber orientation in vivo using 3D ultrasound backscatter tensor imaging. *Sci. Rep.* 7:830. 10.1038/s41598-017-00946-7 28400606 PMC5429761

[B92] Patel M. R. Dai D. Hernandez A. F. Douglas P. S. Messenger J. Garratt K. N. (2014). Prevalence and predictors of nonobstructive coronary artery disease identified with coronary angiography in contemporary clinical practice. *Am. Heart J.* 167 846–852e842. 10.1016/j.ahj.2014.03.001 24890534

[B93] Pathmanathan P. Gray R. A. (2018). Validation and trustworthiness of multiscale models of cardiac electrophysiology. *Front. Physiol.* 9:106. 10.3389/fphys.2018.00106 29497385 PMC5818422

[B94] Plank G. Burton R. A. B. Hales P. Bishop M. Mansoori T. Bernabeu M. O. (2009). Generation of histo-anatomically representative models of the individual heart: tools and application. *Philos. Trans. R. Soc. A Math. Phys. Eng. Sci.* 367 2257–2292. 10.1098/rsta.2009.0056 19414455 PMC2881535

[B95] Potse M. Krause D. Kroon W. Murzilli R. Muzzarelli S. Regoli F. (2014). Patient-specific modelling of cardiac electrophysiology in heart-failure patients. *Europace* 16 (Suppl. 4), iv56–iv61. 10.1093/europace/euu257 25362171 PMC4217520

[B96] Prakosa A. Arevalo H. J. Deng D. Boyle P. M. Nikolov P. P. Ashikaga H. (2018). Personalized virtual-heart technology for guiding the ablation of infarct-related ventricular tachycardia. *Nat. Biomed. Eng.* 2 732–740. 10.1038/s41551-018-0282-2 30847259 PMC6400313

[B97] Quarteroni A. Lassila T. Rossi S. Ruiz-Baier R. (2017). Integrated heart—coupling multiscale and multiphysics models for the simulation of the cardiac function. *Comput. Methods Appl. Mech. Eng.* 314 345–407. 10.1016/j.cma.2016.05.031

[B98] Rice J. J. Wang F. Bers D. M. de Tombe P. P. (2008). Approximate model of cooperative activation and crossbridge cycling in cardiac muscle using ordinary differential equations. *Biophys. J.* 95 2368–2390. 10.1529/biophysj.107.119487 18234826 PMC2517033

[B99] Rodriguez B. Carusi A. Abi-Gerges N. Ariga R. Britton O. Bub G. (2015). Human-based approaches to pharmacology and cardiology: an interdisciplinary and intersectorial workshop. *EP Europace* 18 1287–1298. 10.1093/europace/euv320 26622055 PMC5006958

[B100] Romero D. Sebastian R. Bijnens B. H. Zimmerman V. Boyle P. M. Vigmond E. J. (2010). Effects of the purkinje system and cardiac geometry on biventricular pacing: a model study. *Ann. Biomed. Eng.* 38 1388–1398. 10.1007/s10439-010-9926-4 20094915

[B101] Roney C. H. Williams S. E. Cochet H. Mukherjee R. K. O’Neill L. Sim I. (2018). Patient-specific simulations predict efficacy of ablation of interatrial connections for treatment of persistent atrial fibrillation. *Europace* 20 (Suppl. 3), iii55–iii68. 10.1093/europace/euy232 30476055 PMC6251187

[B102] Rossi S. Lassila T. Ruiz-Baier R. Sequeira A. Quarteroni A. (2014). Thermodynamically consistent orthotropic activation model capturing ventricular systolic wall thickening in cardiac electromechanics. *Eur. J. Mech. A/Solids* 48 129–142. 10.1016/j.euromechsol.2013.10.009

[B103] Ruiz-Baier R. Gizzi A. Rossi S. Cherubini C. Laadhari A. Filippi S. (2014). Mathematical modelling of active contraction in isolated cardiomyocytes. *Math. Med. Biol.* 31 259–283. 10.1093/imammb/dqt009 23760444

[B104] Sainte-Marie J. Chapelle D. Cimrman R. Sorine M. (2006). Modeling and estimation of the cardiac electromechanical activity. *Comput. Struct.* 84 1743–1759. 10.1016/j.compstruc.2006.05.003

[B105] Schulte R. F. Sands G. B. Sachse F. B. Dössel O. Pullan A. J. (2001). Creation of a human heart, model and its customisation using ultrasound images. *Biomed. Eng.* 46 26–28. 10.1515/bmte.2001.46.s2.26

[B106] Scollan D. F. Holmes A. Winslow R. Forder J. (1998). Histological validation of myocardial microstructure obtained from diffusion tensor magnetic resonance imaging. *Am. J. Physiol. Heart Circ. Physiol.* 275 H2308–H2318. 10.1152/ajpheart.1998.275.6.H2308 9843833

[B107] Sebastian R. Zimmerman V. Romero D. Sanchez-Quintana D. Frangi A. F. (2013). Characterization and modeling of the peripheral cardiac conduction system. *IEEE Trans. Med. Imaging* 32 45–55. 10.1109/TMI.2012.2221474 23047864

[B108] Segers P. Stergiopulos N. Westerhof N. (2002). Relation of effective arterial elastance to arterial system properties. *Am. J. Physiol. Heart Circ. Physiol.* 282 H1041–H1046. 10.1152/ajpheart.00764.2001 11834502

[B109] Seo J. H. Abd T. George R. T. Mittal R. (2016). A coupled chemo-fluidic computational model for thrombogenesis in infarcted left ventricles. *Am. J. Physiol. Heart Circ. Physiol.* 310 H1567–H1582. 10.1152/ajpheart.00855.2015 27016582

[B110] Sermesant M. Peyrat J.-M. Chinchapatnam P. Billet F. Mansi T. Rhode K. (2008). Toward patient-specific myocardial models of the heart. *Heart Fail. Clin.* 4 289–301. 10.1016/j.hfc.2008.02.014 18598981

[B111] Shavik S. M. Tossas-Betancourt C. Figueroa C. A. Baek S. Lee L. C. (2020). Multiscale modeling framework of ventricular-arterial bi-directional interactions in the cardiopulmonary circulation. *Front. Physiol.* 11:2. 10.3389/fphys.2020.00002 32116737 PMC7025512

[B112] Shibeshi S. S. Collins W. E. (2005). The rheology of blood flow in a branched arterial system. *Applied* 15 398–405. 10.1901/jaba.2005.15-398 16932804 PMC1552100

[B113] Smith N. de Vecchi A. McCormick M. Nordsletten D. Camara O. Frangi A. F. (2011). euHeart: personalized and integrated cardiac care using patient-specific cardiovascular modelling. *Interface Focus* 1 349–364. 10.1098/rsfs.2010.0048 22670205 PMC3262448

[B114] Sommer G. Schriefl A. J. Andrä M. Sacherer M. Viertler C. Wolinski H. (2015). Biomechanical properties and microstructure of human ventricular myocardium. *Acta Biomater.* 24 172–192. 10.1016/j.actbio.2015.06.031 26141152

[B115] Starobin J. M. Zilberter Y. I. Rusnak E. M. Starmer C. F. (1996). Wavelet formation in excitable cardiac tissue: the role of wavefront-obstacle interactions in initiating high-frequency fibrillatory-like arrhythmias. *Biophys. J.* 70 581–594. 10.1016/S0006-3495(96)79624-8 8789078 PMC1224961

[B116] Streeter Daniel D. Spotnitz Henry M. Patel Dali P. Ross J. Sonnenblick Edmund H. (1969). Fiber orientation in the canine left ventricle during diastole and systole. *Circ. Res.* 24 339–347. 10.1161/01.RES.24.3.339 5766515

[B117] Su B. Zhong L. Wang X. K. Zhang J. M. Tan R. S. Allen J. C. (2014). Numerical simulation of patient-specific left ventricular model with both mitral and aortic valves by FSI approach. *Comput. Methods Prog. Biomed.* 113 474–482. 10.1016/j.cmpb.2013.11.009 24332277

[B118] Sun W. Martin C. Pham T. (2014). Computational modeling of cardiac valve function and intervention. *Annu. Rev. Biomed. Eng.* 16 53–76. 10.1146/annurev-bioeng-071813-104517 24819475 PMC5481457

[B119] Tang D. Yang C. Geva T. Del Nido P. J. (2010). Image-based patient-specific ventricle models with fluid-structure interaction for cardiac function assessment and surgical design optimization. *Prog. Pediatr. Cardiol.* 30 51–62. 10.1016/j.ppedcard.2010.09.007 21344066 PMC3041970

[B120] Tobon-Gomez C. Duchateau N. Sebastian R. Marchesseau S. Camara O. Donal E. (2013). Understanding the mechanisms amenable to CRT response: from pre-operative multimodal image data to patient-specific computational models. *Med. Biol. Eng. Comput.* 51 1235–1250. 10.1007/s11517-013-1044-7 23430328

[B121] Trayanova N. A. (2011). Whole-heart modeling: applications to cardiac electrophysiology and electromechanics. *Circ. Res.* 108 113–128. 10.1161/CIRCRESAHA.110.223610 21212393 PMC3031963

[B122] Trayanova N. A. (2012). Computational cardiology: the heart of the matter. *ISRN Cardiol.* 2012:269680. 10.5402/2012/269680 23213566 PMC3505657

[B123] Trayanova N. A. (2014). Mathematical approaches to understanding and imaging atrial fibrillation: significance for mechanisms and management. *Circ. Res.* 114 1516–1531. 10.1161/CIRCRESAHA.114.302240 24763468 PMC4043630

[B124] Trayanova N. A. Chang K. C. (2016). How computer simulations of the human heart can improve anti-arrhythmia therapy. *J. Physiol.* 594 2483–2502. 10.1113/JP270532 26621489 PMC4850196

[B125] Trayanova N. A. Pashakhanloo F. Wu K. C. Halperin H. R. (2017). Imaging-based simulations for predicting sudden death and guiding ventricular tachycardia ablation. *Circ. Arrhythm. Electrophysiol.* 10:e004743. 10.1161/CIRCEP.117.004743 28696219 PMC5543810

[B126] Vadakkumpadan F. Arevalo H. Prassl A. J. Chen J. Kickinger F. Kohl P. (2010). Image-based models of cardiac structure in health and disease. *Wiley Interdiscip. Rev. Syst. Biol. Med.* 2 489–506. 10.1002/wsbm.76 20582162 PMC2889712

[B127] Vadakkumpadan F. Rantner L. J. Tice B. Boyle P. Prassl A. J. Vigmond E. (2009). Image-based models of cardiac structure with applications in arrhythmia and defibrillation studies. *J. Electrocardiol.* 42:e00151-10. 10.1016/j.jelectrocard.2008.12.003 19181330 PMC2819337

[B128] Valderrabano M. Chen P. S. Lin S. F. (2003). Spatial distribution of phase singularities in ventricular fibrillation. *Circulation* 108 354–359. 10.1161/01.CIR.0000080322.67408.B4 12835210

[B129] Vandervoort Pieter M. Greenberg Neil L. Pu M. Powell Kimerly A. Cosgrove Delos M. Thomas James D. (1995). Pressure recovery in bileaflet heart valve prostheses. *Circulation* 92 3464–3472. 10.1161/01.CIR.92.12.3464 8521568

[B130] Vetter F. J. McCulloch A. D. (1998). Three-dimensional analysis of regional cardiac function: a model of rabbit ventricular anatomy. *Prog. Biophys. Mol. Biol.* 69 157–183. 10.1016/S0079-6107(98)00006-6 9785937

[B131] Vigmond E. J. Clements C. McQueen D. M. Peskin C. S. (2008). Effect of bundle branch block on cardiac output: a whole heart simulation study. *Prog. Biophys. Mol. Biol.* 97 520–542. 10.1016/j.pbiomolbio.2008.02.022 18384847

[B132] Voorhees A. P. Han H.-C. (2015). Biomechanics of cardiac function. *Comprehens. Physiol.* 5 1623–1644. 10.1002/cphy.c140070 26426462 PMC4668273

[B133] Walcott S. Sun S. X. (2009). Hysteresis in cross-bridge models of muscle. *Phys. Chem. Chem. Phys.* 11 4871–4881. 10.1039/B900551J 19506762

[B134] Wall S. T. Walker J. C. Healy K. E. Ratcliffe M. B. Guccione J. M. (2006). Theoretical impact of the injection of material into the myocardium: a finite element model simulation. *Circulation* 114 2627–2635. 10.1161/CIRCULATIONAHA.106.657270 17130342

[B135] Walmsley J. van Everdingen W. Cramer M. J. Prinzen F. W. Delhaas T. Lumens J. (2017). Combining computer modelling and cardiac imaging to understand right ventricular pump function. *Cardiovasc. Res.* 113 1486–1498. 10.1093/cvr/cvx154 28957534

[B136] Wang V. Y. Nielsen P. M. F. Nash M. P. (2015). Image-based predictive modeling of heart mechanics. *Annu. Rev. Biomed. Eng.* 17 351–383. 10.1146/annurev-bioeng-071114-040609 26643023

[B137] Wang W. King M. R. (2012). Multiscale modeling of platelet adhesion and thrombus growth. *Ann. Biomed. Eng.* 40 2345–2354. 10.1007/s10439-012-0558-8 22481228

[B138] Washio T. Okada J. I. Sugiura S. Hisada T. (2012). Approximation for cooperative interactions of a spatially-detailed cardiac sarcomere model. *Cell Mol. Bioeng.* 5 113–126. 10.1007/s12195-011-0219-2 22448201 PMC3291845

[B139] Watson S. R. Dormer J. D. Fei B. (2018). Imaging technologies for cardiac fiber and heart failure: a review. *Heart Fail. Rev.* 23 273–289. 10.1007/s10741-018-9684-1 29500602 PMC6121700

[B140] Weese J. Groth A. Nickisch H. Barschdorf H. Weber F. M. Velut J. (2013). Generating anatomical models of the heart and the aorta from medical images for personalized physiological simulations. *Med. Biol. Eng. Comput.* 51 1209–1219. 10.1007/s11517-012-1027-0 23359255

[B141] Zhang J. M. Zhong L. Luo T. Huo Y. Tan S. Y. Wong A. S. (2014). Numerical simulation and clinical implications of stenosis in coronary blood flow. *Biomed. Res. Int.* 2014:514729. 10.1155/2014/514729 24987691 PMC4058689

[B142] Zhao J. Butters T. D. Zhang H. LeGrice I. J. Sands G. B. Smaill B. H. (2013). Image-based model of atrial anatomy and electrical activation: a computational platform for investigating atrial arrhythmia. *IEEE Trans. Med. Imaging* 32 18–27. 10.1109/TMI.2012.2227776 23192521

[B143] Zhong L. Zhang J.-M. Su B. Tan R. S. Allen J. C. Kassab G. S. (2018). Application of patient-specific computational fluid dynamics in coronary and intra-cardiac flow simulations: challenges and opportunities. *Front. Physiol.* 9:742. 10.3389/fphys.2018.00742 29997520 PMC6028770

